# Spatiotemporal regulation of GSK3β levels by miRNA-26a controls axon development in cortical neurons

**DOI:** 10.1242/dev.180232

**Published:** 2020-02-03

**Authors:** Cristiano Lucci, Raquel Mesquita-Ribeiro, Alex Rathbone, Federico Dajas-Bailador

**Affiliations:** School of Life Sciences, Medical School Building, University of Nottingham, NG7 2UH Nottingham, UK

**Keywords:** GSK3β, Axon, Cortical neurons, miRNA, Polarity

## Abstract

Both the establishment of neuronal polarity and axonal growth are crucial steps in the development of the nervous system. The local translation of mRNAs in the axon provides precise regulation of protein expression, and is now known to participate in axon development, pathfinding and synaptic formation and function. We have investigated the role of miR-26a in early stage mouse primary cortical neuron development. We show that micro-RNA-26a-5p (miR-26a) is highly expressed in neuronal cultures, and regulates both neuronal polarity and axon growth. Using compartmentalised microfluidic neuronal cultures, we identified a local role for miR-26a in the axon, where the repression of local synthesis of GSK3β controls axon development and growth. Removal of this repression in the axon triggers local translation of GSK3β protein and subsequent transport to the soma, where it can impact axonal growth. These results demonstrate how the axonal miR-26a can regulate local protein translation in the axon to facilitate retrograde communication to the soma and amplify neuronal responses, in a mechanism that influences axon development.

## Abstract (precis)

**Highlighted Article:** Axonal miR-26a can regulate GSK3β translation in the axon to promote retrograde communication to the soma in a mechanism that modulates axon development.

## INTRODUCTION

The morphological polarisation of pyramidal neurons through the development of multiple dendrites and a long axonal projection is one of the most complex structural and functional challenges faced by any cell type. The mechanisms, both cellular and molecular, that control this process have been extensively investigated, leading to the identification of many important intracellular signalling pathways and molecules, including phosphoinositide 3-kinase (PI3K), Ras homologous (Rho)-GTPase, Par3/6, mammalian target of rapamycin (mTOR) and protein kinase A (PKA) (Arimura and Kaibuchi, 2007; Barnes and Polleux, 2009; Hapak et al., 2018). Among these, glycogen synthase kinase 3β (GSK3β) has been positioned at a signalling crossroads where it coordinates the complex emergence of axon/dendrite morphology in neurons (Kim and Snider, 2011).

GSK3 proteins are serine/threonine kinases that have been described as key regulators across multiple neurodevelopmental processes, from neurogenesis, neuronal migration and neuronal polarisation, to axon growth and guidance (Hur and Zhou, 2010; Kim and Snider, 2011). This broad regulatory capacity of GSK3 proteins can be explained by its long list of functional substrates, including transcription factors such as cyclic AMP response element-binding protein (CREB), neurogenin 2, Smad1, Jun and β-catenin (Hur and Zhou, 2010). Moreover, the targeting of transcription factors is complemented by the ability of GSK3 to control the activity of several microtubule-associated proteins, such as Map1b and Tau (Kim and Snider, 2011; Kim et al., 2006; Zhou and Snider, 2005). For example, moderate inactivation of GSK3 at the growth cone can promote microtubule stability, which is necessary for efficient axon regeneration (Hur et al., 2011).

Unlike many other kinases, GSK3 proteins are normally active in resting cells, with several regulatory mechanisms controlling their activity, including protein-protein interactions, spatial regulation and phosphorylation (Etienne-Manneville and Hall, 2003; Hengst et al., 2009; Thornton et al., 2008; Wu et al., 2009). The constitutive activity, together with the complex array of post-translational mechanisms that can control substrate specific actions (Beurel et al., 2015), suggest the need for tight regulatory mechanisms that can control GSK3 levels. Despite this, the protein levels of GSK3β during the establishment of neuronal polarity do not appear to increase after inhibition of the proteasome, suggesting a lack of proteasome regulation (Yan et al., 2006) and highlighting the potential need and importance of GSK3β translation regulation.

Local translation of mRNAs in the axon allows the precise temporal and spatial regulation of protein expression (Cioni et al., 2018; Costa and Willis, 2018). Rather than just a distinctive characteristic of developmental processes, axonal protein synthesis is now considered an integral part of the biology of a neuron, participating in processes needed for development, growth, pathfinding, and formation and maintenance of pre-synaptic terminals (Batista et al., 2017; Campbell and Holt, 2001; Deglincerti et al., 2015; Gracias et al., 2014; Hengst et al., 2009; Hengst and Jaffrey, 2007; Jung et al., 2012; Piper et al., 2006; Sasaki et al., 2010; Yao et al., 2006).

In addition to the control of protein content in the axon compartment, local protein synthesis offers the possibility of communication from the axon to the neuronal soma (Batista and Hengst, 2016). In effect, neurotrophins have been shown to signal retrogradely in peripheral neurons after triggering the local translation of specific effectors and/or transcription factors (Cox et al., 2008; Ji and Jaffrey, 2012; Willis et al., 2007). In injury models of the peripheral nervous system, local translation of importin β1 and α3, together with RanBP1 are used in the retrograde signalling complex that delivers the injury signal to the soma (Hanz et al., 2003; Yudin et al., 2008). Recently, a similar mechanism of information transmission was observed for hippocampal neurons, where an axonal insult by Aβ_1-42_ triggered the local translation of sentinel mRNAs encoding components of a retrograde signalling complex that modulate the soma response to Aβ_1-42_ (Walker et al., 2018). Although ‘axon to soma’ retrograde signalling after local translation has been described in response to injury insults, evidence of its occurrence in developing axons, particularly in the CNS, is absent.

The mRNA transcripts that are transported along the axon are highly heterogeneous, being sensitive to both developmental cues and pathophysiological conditions (Costa and Willis, 2018), which has prompted the investigation of novel regulatory mechanisms (Jung et al., 2012). Among those described so far, miRNAs have emerged as important players in multiple cellular processes, such as neurogenesis, axon development, pathfinding and neuron connectivity (Bellon et al., 2017; Dajas-Bailador et al., 2012; Hancock et al., 2014; Kaplan et al., 2013; Reh and Hindges, 2018). Despite this growing number of studies demonstrating the importance of miRNAs in axon and synapse development (Rajman and Schratt, 2017; Swanger and Bassell, 2011), evidence for their role in axon specification and neuronal polarisation has been largely missing. Only recently, miR-338, which was previously reported to control axonal outgrowth in cortical and superior cervical ganglion neurons (Aschrafi et al., 2008; Kos et al., 2017b), was shown to modulate cortical neuron migration and to have an effect in neuronal morphology and polarity *in vivo* (Kos et al., 2017a). Furthermore, a recent paper by Ambrozkiewicz et al. (2018) demonstrated the capacity of miR-140 to act synergistically with its host gene E3 ubiquitin ligase WW-containing protein 2 (*Wwp2*) and *Wwp1* in the establishment of axon-dendrite polarity of developing cortical neurons *in vivo*.

Among the many miRNAs known to participate in neuron development, the miR-26 family (miR-26a-1, miR-26a-2 and miR-26b) have a known role in tissue growth and differentiation, with regulated expression during development and tumorigenesis (Gao and Liu, 2011). In the nervous system, miR-26a is highly expressed in the mouse cerebral cortex at embryonic day 12 and throughout cortical development, where it has been shown to regulate neural progenitor differentiation and cell-cycle progression (Lambert et al., 2018; Zhang et al., 2018). Beyond this role in differentiation, the knocking down of miR-26a in peripheral sensory neurons led to impaired axon regeneration (Jiang et al., 2015). In a brief report that uncommonly used rat neonatal cortical neuron cultures for neurite growth assays, inhibition of miR-26a showed an effect in neuritic/dendritic growth, via the targeting of phosphatase and tensin homologue (PTEN) (Li and Sun, 2013).

Considering that both GSK3β and PTEN are involved in pathways that control neuron polarisation processes, we decided to investigate the role of miR-26a in axon specification and polarity using mouse primary cortical neurons, a model that provides the molecular and cellular accessibility needed to dissect these processes. We show that miR-26a is highly expressed during the development of primary cortical cultures and regulates both axon specification and growth. Importantly, the use of compartmentalised microfluidic chambers allowed us to reveal a local role for miR-26a in the regulation of axon development, in a process that requires the repression of local synthesis of GSK3β in the axon. Removal of miR-26a-mediated repression in the axon, triggers the local translation of GSK3β protein and its subsequent transport to the neuronal soma, where its activity can further regulate axonal function.

## RESULTS

### miR-26a regulates neuron polarisation and axonal growth in cortical primary neurons

**Fig. 1.**
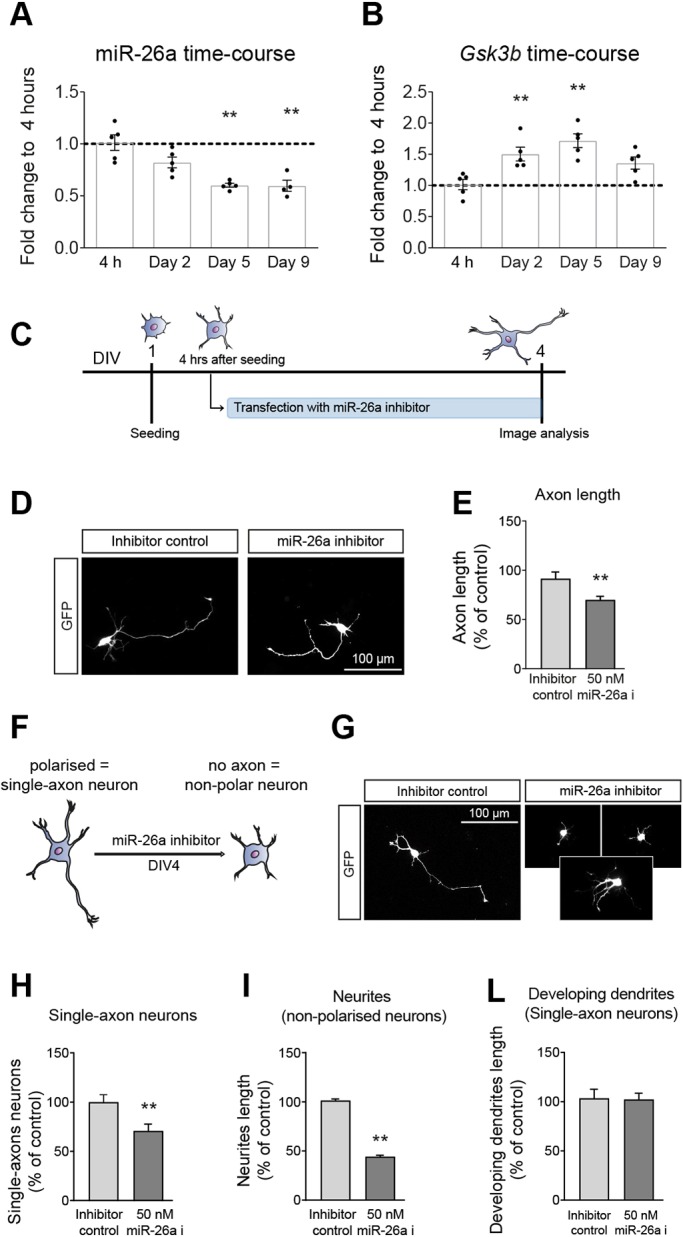
**miR-26a is expressed in primary cortical neurons and regulates neuronal polarisation and axonal outgrowth*.*** (A) Quantification of miR-26a levels over development of cortical primary cultures from 4 h to 9 days *in vitro*. Expression of miR-26a-5p was analysed by relative quantification using the comparative Ct method (2^−ΔΔCt^) and the geometric mean of miR-100-5p, miR-128-3p, miR-134-5p, miR-434-3p and let7a-5p was used as a reference; data are mean±s.e.m. of five independent experiments. (B) Quantification of *Gsk3b* expression levels over development of cortical primary cultures from 4 h to 9 days *in vitro*. Expression of *Gsk3b* was analysed by relative quantification using the comparative Ct method (2^−ΔΔCt^). The geometric mean of *Gapdh* and *Ube2* was used as a reference; mean±s.e.m. of five independent experiments. (C) Diagrammatic representation of the experimental design used in D-L. (D) Representative images of polarised cortical neurons used for axon length measurements after transfection with GFP plus a miR-26a inhibitor. (E) Quantification of axon length after inhibition of miR-26a (50 nM miR-26a i), showing up to a 25% decrease compared with a non-targeting control, *n*=5. (F,G) Schematic representation and images of polarity changes induced by an miR-26a inhibitor on cortical neurons. (H) Quantification of the number of single-axon neurons after inhibition of miR-26a, expressed as a percentage of neurons transfected with non-targeting control, *n*=5. (I) Quantification of the length of all neurites from non-polarised neurons, *n*=5. (L) Quantification of developing dendrites in single-axon neurons, *n*=5. Data are mean±s.e.m.; one-way ANOVA with Bonferroni's multiple comparison post-hoc tests (A,B). Student's *t*-test (E-L), **P≤0.01.

To investigate the role of miR-26a in the development of CNS neurons *in vitro*, we first evaluated its expression in mouse primary cortical neuron cultures. We found miR-26a being expressed in cortical neurons at 4 h post plating, with an early trend towards decrease that stabilises in more developed cultures up to 9 days *in vitro* (Fig. 1A). Levels of the miR-26a target *Gsk3b* showed the opposite progression (Fig. 1B).

Inhibition of miR-26a generated a significant decrease in the axonal length of cortical primary neurons, similar to findings in peripheral sensory axons and neurites (Jiang et al., 2015; Li and Sun, 2013). In our studies with cortical neurons, transfection was carried out 4 h after plating, and their development was evaluated 72 h later (Fig. 1C). At this timepoint, inhibition of miR-26a significantly decreased axonal length compared with non-targeting control probes (Fig. 1D,E). However, a closer morphological examination of the transfected primary cortical cultures also revealed a significant reduction in the proportion of neurons with a single-axon phenotype (i.e. those polarised with a distinct and unique axon projection; Fig. 1F-H). Although the total length of neurites in non-polarised neurons was significantly decreased (Fig. 1I), in those neurons with a single differentiated axon, the rest of the developing dendrites did not show any change in length after inhibition of miR-26a (Fig. 1L).

**Fig. 2.**
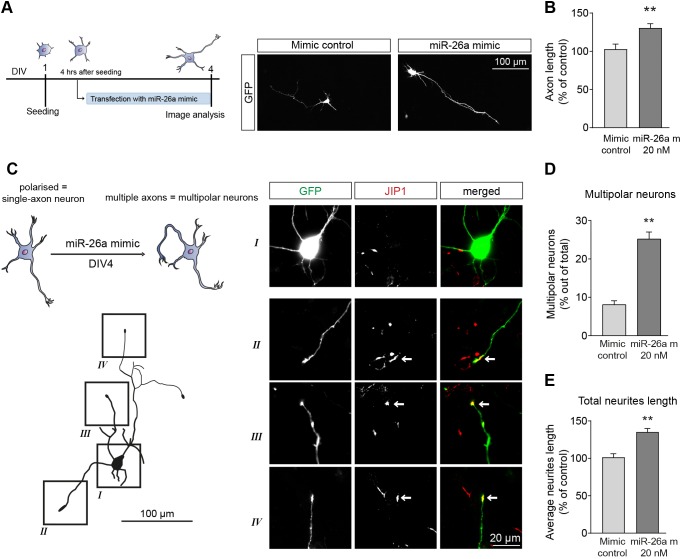
**miR-26a overexpression induces axonal outgrowth and formation of multiple axon-like processes*.*** (A) Diagrammatic representation of the experimental design used in B-E and representative images of cortical neurons used for axon length measurements after transfection with GFP plus the miR-26a mimic. (B) Quantification of axon length in single-axon neurons after overexpression of miR-26a (miR-26a m 20 nM), showing an increase in axon length up to almost 40% compared with a non-targeting control, *n*=8. (C) Schematic representation and trace to demonstrate the polarity changes induced by the miR-26a mimic, showing the increase in the number of neurons with multiple axons. Squares (I-IV) on the trace correspond to images from soma and neurite terminals of cortical neuron. Arrows indicate JIP1 labelling, which was used as a marker of axonal growth cones. (D) Quantification of the number of neurons with multiple axon-like processes after overexpression of miR-26a and expressed as a percentage of total neuron number transfected, *n*=5. (E) Quantification of the overall length of all projecting neurites in multipolar neurons, *n*=5. Data are mean±s.e.m. Student's *t*-test, **P≤0.01.

To further investigate the potential role of miR-26a in neuron polarisation, we carried out overexpression studies using a miR-26a mimic. The rise in miR-26a levels in cortical neurons produced a significant increase in axonal growth (Fig. 2A,B). Crucially, transfection with the miR-26a mimic also induced an increase in the number of neurons with multiple axon-like processes (Fig. 2C,D), identified by the presence of the axonal marker JIP-1 (Dajas-Bailador et al., 2014, 2008; Deng et al., 2014; Fu and Holzbaur, 2013), and by the more traditional axonal marker tau (Fig. S1C,D). This multi-polar neuronal phenotype was accompanied by a general increase in the length of all projections, which were ∼40% longer than the total average length of all neurites in same stage control cultures (Fig. 2E). Overall, these results demonstrate that, in addition to affecting axonal growth in cortical neurons, miR-26a can influence neuronal polarity.

The capacity of miR-26a to modulate axon specification and growth, two cellular processes that are intrinsically linked in early stage neuron polarisation, made us speculate whether miR-26a could retain the ability to control both neuronal polarity and growth at different stages of neuron development in culture. To assess this, we transfected cortical neurons at two further time-points (24 h and DIV5) with either miR-26a inhibitor or mimic, and analysed the ability to develop/maintain polarity 72 h later. When transfected at 24 h, inhibition of miR-26a still decreased both axonal growth and the number of single-axon neurons (Fig. S2A-C). However, we found no significant changes in the polarisation of cortical neurons when the activity of miR-26a was inhibited at the later time point (DIV5). Unlike the effect seen at 24 h, transfection of the miR-26a inhibitor after 5 days of culture did not decrease the percentage of single-axon neurons (Fig. S2D,E). More importantly, even the overexpression of the microRNA at this later time point did not significantly increase the number of neurons with multiple axons (Fig. S2F,G).

### miR-26a regulates the expression levels of GSK3β protein in primary cortical neurons

The search for a molecular mechanism that elucidates the capacity of miR-26a to control both neuron polarity and axon outgrowth led us to investigate PTEN and GSK3β, which, respectively, bear three and two highly conserved miR-26a-binding site sequences in their 3′UTRs (Fig. S3A,B) and have been previously described as functional targets of miR-26a (Cui et al., 2015; Jiang et al., 2015; Li and Sun, 2013). Knockdown of GSK3β and the use of specific inhibitors cause the formation of multiple axons (Gartner et al., 2006; Jiang et al., 2005). As depicted by Jiang et al. (2005), GSK3β manipulations prevail over PTEN on neuronal polarity, indicating that PTEN acts upstream of GSK3β in polarity formation. For this reason, we decided to initially focus on the latter and test our hypothesis of whether GSK3β is a functional target of miR-26a in cortical primary neurons.

Taking advantage of the morphological polarisation of cortical neurons *in vitro*, we investigated whether miR-26a can directly regulate the expression levels of GSK3β protein in neuronal somas and/or growth cones by quantitative immunostaining. Overexpression of miR-26a decreased GSK3β levels in both the soma and axonal growth cones (Fig. S4A-C). Inhibition of miR-26a raised the levels of the GSK3β protein in both morphological regions, but only in those neurons that had managed to develop a growing axon (Fig. S4D,E). Instead, in those neurons that failed to develop an axon, addition of miR-26a inhibitor did not increase GSK3β levels in the soma (data not shown). The examination of GSK3β levels following inhibition of miR-26a in culture conditions was previously reported after electroporation of mouse sensory neurons, in a process that did not address specific subcellular localisation (Jiang et al., 2015). Here, analysis of global protein levels in primary cortical neurons using western blotting did not show significant changes (data not shown), likely reflecting a sub-optimal incorporation of the inhibitor and mimic. To test this, we used the highly transfectable neuroblastoma cell line neuro-2A (N2A) in experiments with overexpression and inhibition of miR-26a, which demonstrated the expected decrease and increase in GSK3β (Fig. S4F,G). At the subcellular level, our immunofluorescence imaging studies in primary cortical neurons demonstrate that the effect of miR-26a on GSK3β levels could be specifically detected in the axon growth cones.

This is an important observation as it may suggest a local effect of this microRNA in the axon compartment, as previously reported with other microRNAs (Bellon et al., 2017; Dajas-Bailador et al., 2012; Hancock et al., 2014; Zhang et al., 2015). The capacity for miRNAs to regulate axon development, and to do so by localising to the axon compartment is a relatively new area of investigation (Bellon et al., 2017; Dajas-Bailador et al., 2012; Wang and Bao, 2017). To address whether this potential mechanism was relevant in the effects observed for miR-26a, we first assessed the presence of both miR-26a and *Gsk3b* mRNA in the axons of cortical primary neurons. For this, we cultured neurons in compartmentalised microfluidic chambers, which allow the morphological and functional separation of axons from somas (Taylor et al., 2005). Both miR-26a and *Gsk3b* mRNA are detected in axonal RNA by RT-qPCR (see Materials and Methods; Poulopoulos et al., 2019).

### GSK3β mediates the functional effects of miR-26a in neuron polarisation and growth

**Fig. 3.**
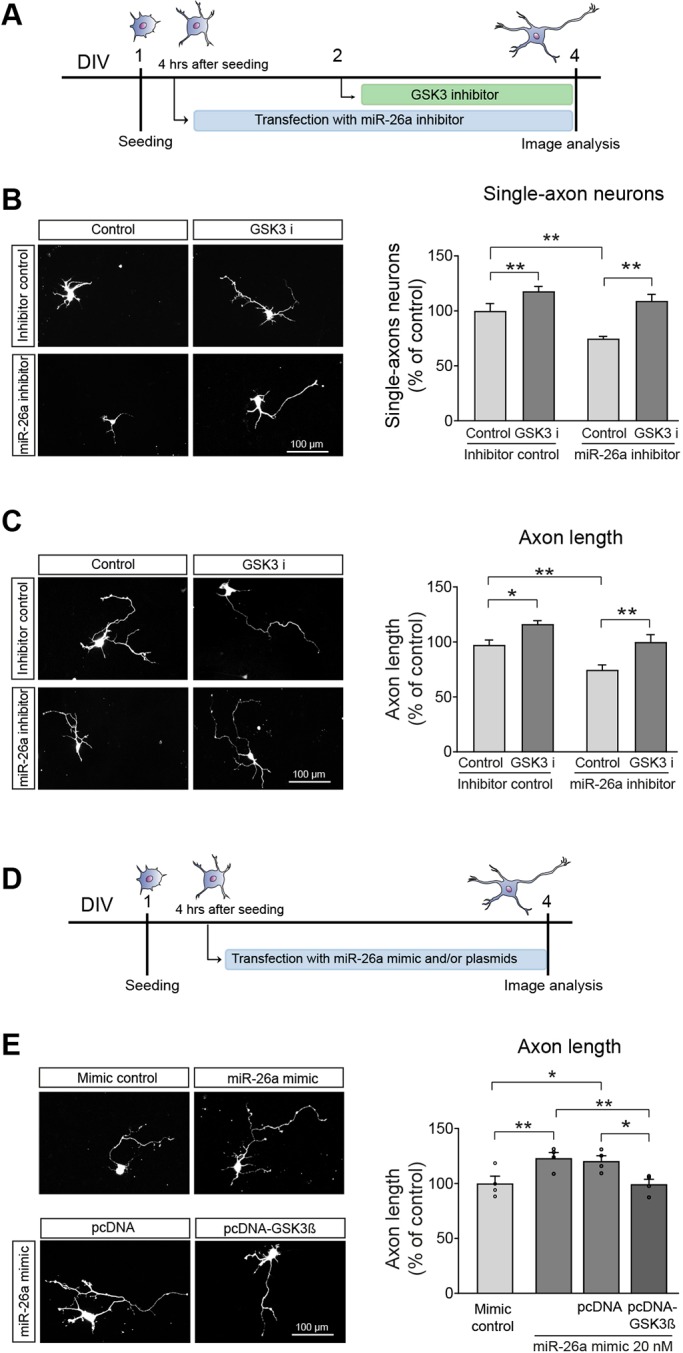
**GSK3β mediates the functional effects of miR-26a in neuron polarisation and growth*.*** (A) Diagrammatic representation of the experimental design used in B and C. (B) Representative images and quantification of the number of single-axon neurons after inhibition of miR-26a and the addition of GSK3 inhibitor (SB415286, 1 µM) 24 h after transfections, *n*=7. (C) Representative images and quantification of axon length after inhibition of miR-26a and the addition of GSK3 inhibitor (SB415286, 1 µM) 24 h after transfections, *n*=7. (D) Diagrammatic representation of the experimental design used in E. (E) Representative images and quantification of axon length after overexpression of both miR-26a and GSK3β, *n*=4. Data are mean±s.e.m. one-way ANOVA with Bonferroni's multiple comparison post-hoc tests, *P<0.05, **P≤0.01.

**Fig. 4.**
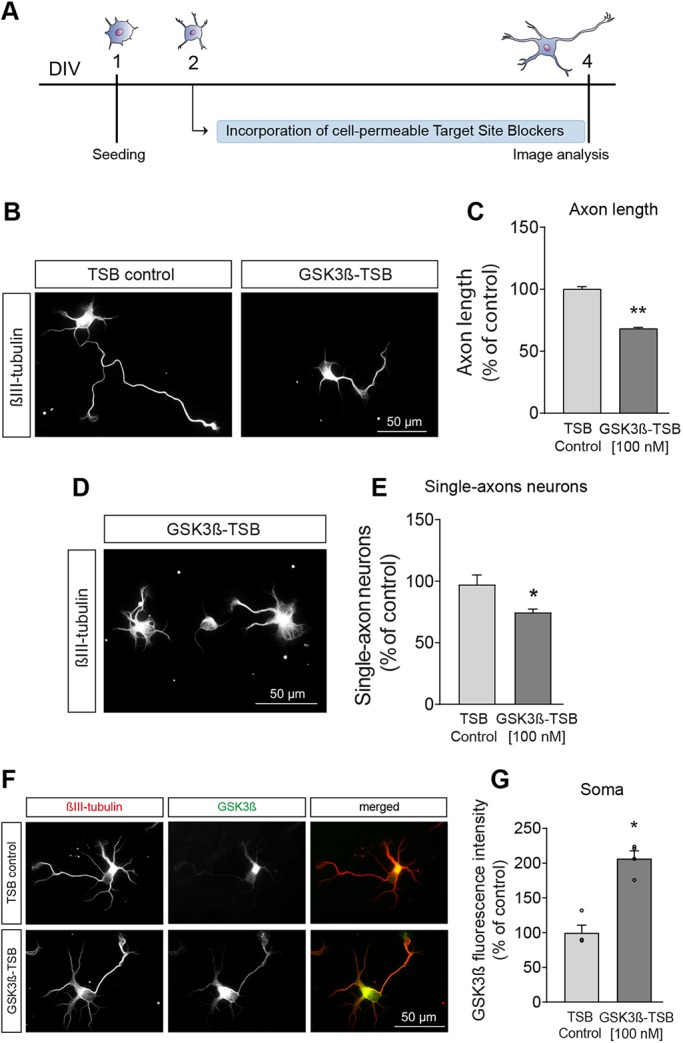
**Block of GSK3β targeting by miR-26a recapitulates effects on neuron polarisation and axon length.** (A) Diagrammatic representation of the experimental design used in B-G. (B) Representative images of neurons after the addition of GSK3β-TSB oligos (GSK3β-TSB, 100 nM), *n*=5. (C) Quantification of axon length after addition of GSK3β-TSB and respective controls (TSB control, 100 nM), *n*=5. (D,E) Representative image and quantification of single-axon neurons after addition of GSK3β-TSB, *n*=4. (F,G) Representative images and quantification of GSK3β protein levels expressed as a percentage of respective controls, *n*=4. Data are mean±s.e.m. Student's *t*-test, *P<0.05, **P≤0.01.

Considering the capacity of miR-26a to control GSK3β protein levels in primary cortical neurons, we evaluated its role in functional rescue experiments. For this, we employed two experimental approaches. One was the use of a pharmacological inhibitor of GSK3 activity (SB415286), which has been extensively used in the past (Coghlan et al., 2000; Gobrecht et al., 2014; Guo et al., 2017; Kim et al., 2006; Yoshimura et al., 2005). In our cultures, inhibition of GSK3 activity increased both axonal length and the percentage of single-axon neurons (Fig. S5A-C) (Jiang et al., 2005; Yoshimura et al., 2005). The second approach was to use a GSK3β plasmid in overexpression studies. As shown in Fig. S5D-F, transfection of cortical neurons with pcDNA-GSK3β decreased axonal length and the percentage of single-axon neurons. Overall, these two experimental approaches confirmed the role of GSK3β in the specification and growth of axons in cortical neurons, and allowed us to attempt functional rescue experiments after the inhibition and overexpression of miR-26a. Pharmacological inhibition of GSK3 activity reversed the effect of the miR-26a inhibitor with regards to neuronal polarity. For this, transfections were again performed at 4 h after plating, with SB415286 (1 µM) being added 24 h after transfections. Lack of GSK3 activity abolished the drop in 23% of single-axon neurons after inhibition of miR-26a, returning to those seen in control conditions (Fig. 3A,B). The effect of pharmacological inhibition of GSK3 was not restricted to polarity; it also reverted the decrease in axon length after inhibition of miR-26a (Fig. 3C). Conversely, we found that overexpression of GSK3β counterbalanced the increase in axon length after transfection with miR-26a mimic (Fig. 3D-E). As the overexpression of PTEN also compensated the rise in axon outgrowth after overexpression of miR-26a (Fig. S5G-L), it was important to define the main effector in our experimental model. For this, we designed target site blocker oligos (GSK3β-TSB), which specifically prevent the capacity of miR-26a to bind with GSK3β, without affecting other target interactions, including PTEN. Addition of this GSK3β-TSB produced both a decrease in axonal length and in the number of single-axon neurons (Fig. 4A-E), similar to the effects seen with the miR-26a inhibitor. Moreover, levels of GSK3β protein also increased after incubation with the GSK3β-TSB (Fig. 4F,G). Together, these results indicate that miR-26a can control neuronal polarity and axon outgrowth through a mechanism that is mainly dependent on GSK3β levels and activity.

### Localised inhibition of miR-26a in the axon can regulate axonal growth via GSK3β signalling

The use of primary cortical neurons in culture provides an opportunity for the study of cellular mechanisms with great spatiotemporal detail. It was thus important to determine whether the capacity to control polarity and growth is achieved via changes in GSK3β expression in specific subcellular domains.

**Fig. 5.**
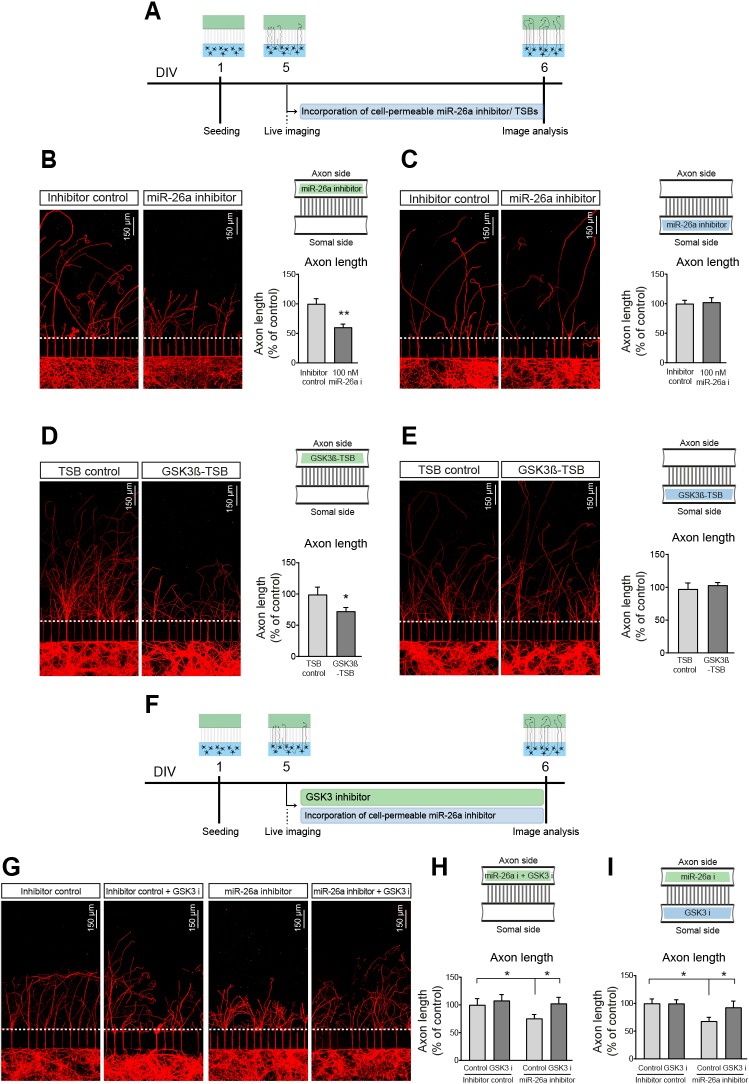
**Local miR-26a effects in the axon can regulate axonal growth via GSK3β signalling.** (A) Diagrammatic representation of the experimental design used in B-E. (B) Representative images and quantification of axonal length after using a cell-permeable inhibitor of miR-26a specifically applied to the axon side of microfluidic chambers, *n*=8. (C) Representative images and quantification of axonal length after using a cell-permeable inhibitor of miR-26a specifically applied to the somal side of microfluidic chambers, *n*=6. (D) Representative images and quantification of axonal length after using a cell-permeable GSK3β-TSB (100 nM) specifically applied to the axon side of microfluidic chambers, *n*=6. (E) Representative images and quantification of axonal length after using a cell-permeable GSK3β-TSB specifically applied to the somal side of microfluidic chambers, *n*=5. (F) Diagrammatic representation of the experimental design used in G-I. (G) Representative images and (H) quantification of axonal length after using both miR-26a and GSK3 inhibitor (SB415286, 1 µM) applied to the axonal side of microfluidic chambers, *n*=8. (I) Quantification of axonal length after application of miR-26a in the axon side and GSK3 inhibitor in the somal side of microfluidic chambers, *n*=8. For all panels, schematics of the microfluidic chambers (upper right corner) depict where drugs were added. Data are mean±s.e.m. Student's *t*-test (B-E), Kruskal–Wallis with Dunn's multiple comparison test (H), one-way ANOVA with Bonferroni's multiple comparison post-hoc tests (I), *P<0.05, **P≤0.01.

For this, we used compartmentalised microfluidic chambers, which allow the selective application of cell permeable miRNA inhibitors, siRNA probes and/or drug treatments to either compartment (Dajas-Bailador et al., 2012; Hengst et al., 2009; Taylor et al., 2005; Zhang et al., 2015). Neurons were seeded in the soma side and grown for 5-6 days in order to allow a significant number of axons to cross into the axonal side of the device (Fig. S6A). At this point, the cell-permeable inhibitor of miR-26a was added to either the soma or axon compartment of the chambers. The length of the axons was established from the site at which they entered the axonal side of chambers up to the growth cone and monitored for 48 h after application (Fig. S6A). As depicted in Fig. S6B and Fig. 5A,B, axonal outgrowth is drastically reduced when the miR-26a inhibitor is applied exclusively in the axonal side of microfluidic chambers. Importantly, this effect on axonal growth is not observed when the miR-26a inhibitor was added to the soma side (Fig. S6C and Fig. 5C). The GSK3β-TSB for miR-26a produced a similar outcome to that of the miR-26a inhibitor, both in the axon and soma side (Fig. 5D-E). These data demonstrate that the effect on axonal growth requires a local mechanism triggered in the developing axons of cortical neurons.

In this context, we reasoned that axonal application of the inhibitor of GSK3 activity would rescue the locally mediated decrease in axon growth induced by inhibition of miR-26a, which should increase GSK3β expression. As predicted, addition of SB415286 into the axonal compartment abolished the decrease in axonal growth mediated by the miR-26a inhibitor (Fig. 5F-H). However, unlike the experiments with miR-26a inhibitor alone, which failed to affect axonal growth when applied to the soma, the inhibition of GSK3 activity in the soma side also rescued the decrease in axon length observed after axonal inhibition of miR-26a function (Fig. 5I).

The observation that GSK3β activity in the soma is necessary to prevent the effect of miR-26a inhibition on axon growth is in agreement with previous findings in peripheral neuron regeneration (Jiang et al., 2015). However, our results provide further mechanistic insight and suggest that a local effect of miR-26a present in the axon is required for the control of axon growth in cortical neurons. These set of results put forward the interesting possibility that local translation of GSK3β in the axon is a pre-requisite for function. Newly synthesised GSK3β may then act on axon-dependent mechanisms, likely impacting on cytoskeletal dynamics, but also undergo retrograde transport towards the soma where it activates further regulatory mechanisms controlling axonal growth. This retrograde transport of locally synthesised proteins as a mechanism of axonal signalling has been only demonstrated for a small number of transcription factors (Cox et al., 2008; Ji and Jaffrey, 2012; Willis et al., 2007), and very recently in a neurodegenerative CNS neuronal model (Walker et al., 2018). However, this functional mechanism had not been shown for intra-axonal miRNA-regulated translation.

### Retrograde transport of locally translated GSK3β is required for the regulation of axonal growth after inhibition of miR-26a in the axon

**Fig. 6.**
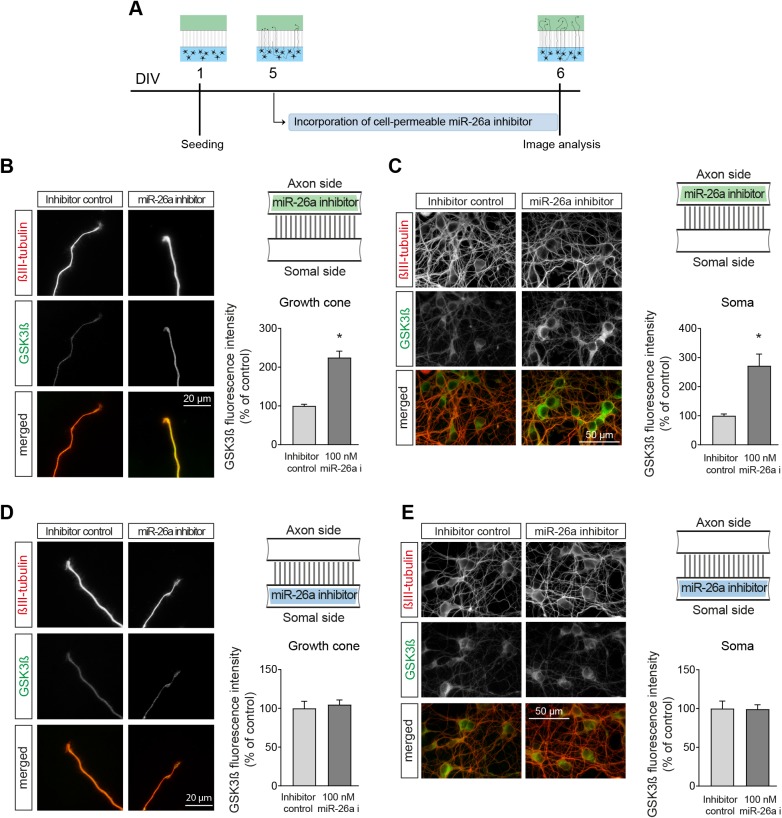
**Inhibition of miR-26a in the axon increases the expression levels of GSK3β protein in neuronal somas and axon growth cones.** (A) Diagrammatic representation of the experimental design used in B-E. (B,C) Representative images and quantification of GSK3β protein levels in the (B) growth cones and (C) somas of cortical neurons after local application of cell-permeable miR-26a inhibitor in the axon side of microfluidic chambers, *n*=8. (D,E) Representative images and quantification of GSK3β protein levels in the (D) growth cones and (E) somas of cortical neurons after local application of cell-permeable miR-26a inhibitor in the somal side of microfluidic chambers, *n*=5. Data are mean±s.e.m. Student's *t*-test, *P<0.05.

To test our hypothesis, we first evaluated the levels of GSK3β protein in the axon and soma of cortical neurons after compartmentalised application of the miR-26a inhibitor. Inhibition of miR-26a alone in the axon compartment of microfluidic chambers produced a significant increase in GSK3β protein levels, both in the axon growth cone and soma of cortical neurons (Fig. 6A-C). Application of the miR-26a inhibitor to only the soma side failed to produce an increase in GSK3β protein, both in the soma and axonal growth cones (Fig. 6D,E). As independent controls of the immunostaining protocol, Fig. S7A-D data show the lack of change in βIII-tubulin levels after both experimental conditions. Overall, these findings support the idea that miR-26a can regulate GSK3β levels in the axon, but to achieve its full functional effect, it requires an increase in GSK3β levels in the soma that is axon dependent.

**Fig. 7.**
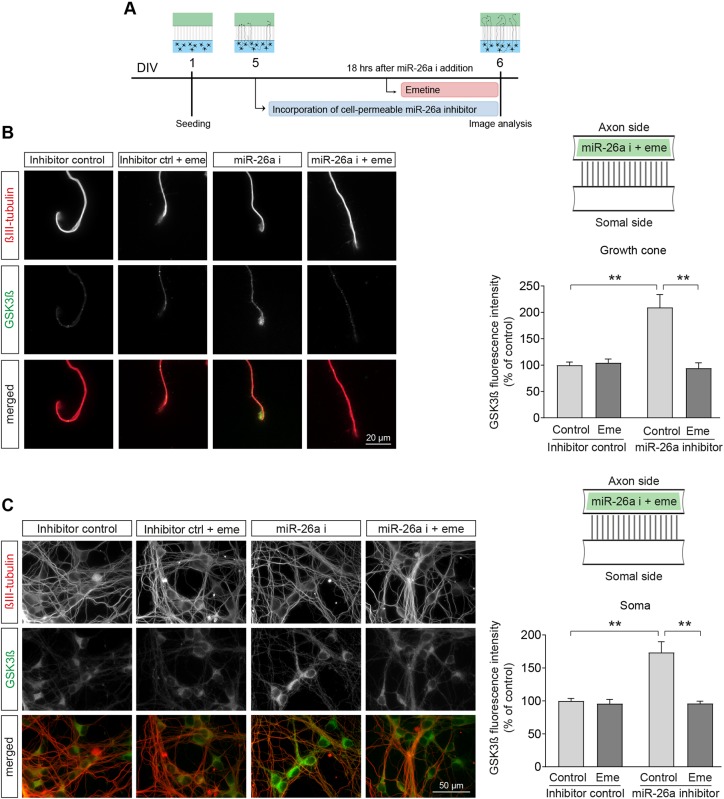
**Inhibition of protein translation in the axon prevents the increase in GSK3β levels in both axon and soma subcellular compartments.** (A) Diagrammatic representation of the experimental design used in B and C. (B,C) Representative images and quantification of GSK3β protein levels in both (B) growth cones and (C) somas of cortical neurons treated with translation inhibitor emetine (100 nM) 18 h after axonal application of miR-26a inhibitor and imaged 6 h later. For all the panels, schematics of the microfluidic chambers (above the graphs) depict where drugs were added. Applications to the axon side are illustrated in green, *n*=5. Data are mean±s.e.m.; one-way ANOVA with Bonferroni's multiple comparison post-hoc tests, **P≤0.01.

Direct demonstration that miR-26a regulates GSK3β translation in the axons was obtained following the addition of the translation blocker emetine (Villarin et al., 2016) in the axon compartment after local inhibition of miR-26a. In this experiment, cortical axons were treated with emetine 18 h after axonal application of miR-26a inhibitor and imaged 6 h later. As seen in Fig. 7A-C, emetine addition prevented the increase in GSK3β levels in both the axon and soma of cortical neurons. This experiment confirms that translation in the axon is needed for the observed increase in GSK3β levels in both subcellular domains.

To further examine the validity of this novel mechanism, we devised a compartmentalised culture model where overall axonal transport is impaired, thus preventing effective retrograde signalling of axonally synthesised GSK3β. For this, we used a microtubule-destabilising drug, nocodazole, which has been previously demonstrated to disrupt axonal transport (Gobrecht et al., 2014; Saijilafu et al., 2013; Twelvetrees et al., 2016), an observation that we further confirmed in our own experimental model using live imaging of mitochondria (Movies 1 and 2). In this experiment, addition of nocodazole significantly decreased the number of moving mitochondria, while increasing those in static condition (Fig. S8A).

**Fig. 8.**
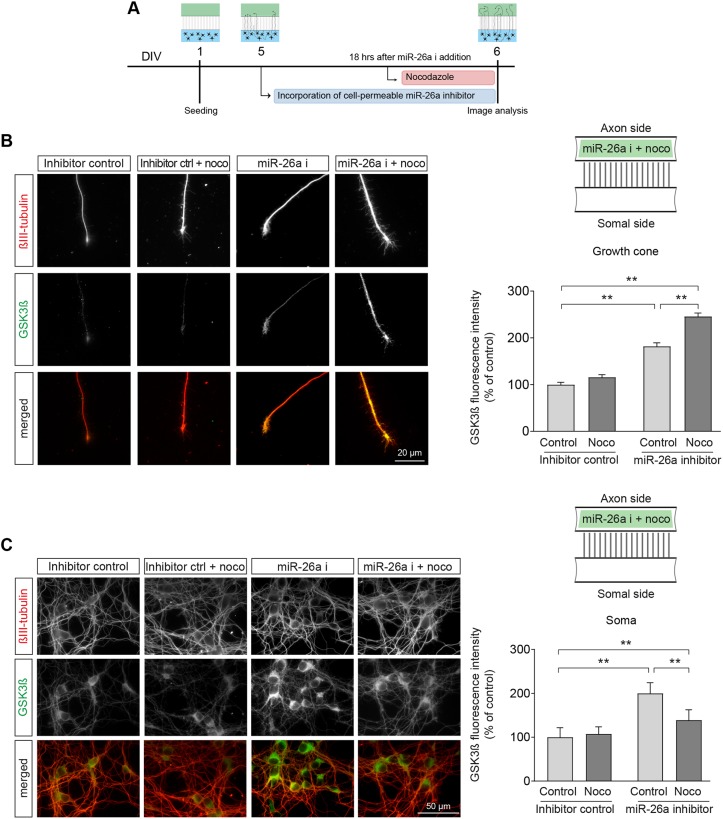
**Retrograde transport of locally translated GSK3β after inhibition of miR-26a in the axon.** (A) Diagrammatic representation of the experimental design used in B and C. (B,C) Representative images and quantification of GSK3β protein levels in both (B) growth cones and (C) somas of cortical neurons treated with nocodazole (100 nM) 18 h after axonal application of miR-26a inhibitor and imaged 6 h later. For all the panels, schematics of the microfluidic chambers (above the graphs) depict where drugs were added. Applications to the axon side are illustrated in green, *n*=8. Data are mean±s.e.m.; one-way ANOVA with Bonferroni's multiple comparison post-hoc tests, **P≤0.01.

Following this corroboration of impairment of axonal transport, cortical axons were treated with nocodazole 18 h after axonal application of miR-26a inhibitor and imaged 6 h later. As shown in Fig. 8A,B, addition of nocodazole after inhibition of miR-26a in the axon still led to a significant increase in axonal GSK3β levels, but, crucially, prevented the previously observed increase in the soma (Fig. 8C). Overall, when these experiments are put together with our local application of emetine and functional studies, they provide demonstration of two key mechanisms. First, local synthesis of GSK3β in the axon is regulated by axonal miR-26a, which is normally repressing its translation. Second, regulation of axonal growth by GSK3β after release of miR-26a repression requires the transport of newly synthesised GSK3β to the somas of cortical neurons.

## DISCUSSION

The tight control of multiple signalling pathways allows the development of axon/dendrite polarity in neurons and provides the structural platform for the establishment of neuronal communication in the nervous system (Barnes and Polleux, 2009; Namba et al., 2015). Here, we demonstrate how a single microRNA can modulate two distinct but also sequentially related cellular processes, axon specification and growth, by controlling the axonal translation of GSK3β. In primary cortical neurons, inhibition of endogenous miR-26a leads to a remarkable process of long-distance signalling, where both local axon translational of GSK3β and its transport to the soma are required for the regulation of axon development.

The capacity for miR-26a to regulate these neuronal processes supports previous experimental evidence that axonal growth is not just a consequence of axonal specification (Jiang et al., 2005). As such, axon ablation to eliminate length differences can reset polarity (Bradke and Dotti, 2000; Dotti and Banker, 1987), while promotion of neurite growth can lead to axon specification (Lamoureux et al., 2002; Nakamuta et al., 2011). Although axon specification versus growth is the focus of active study, research has also shown that several molecules, including GSK3β, can regulate both processes (Arimura and Kaibuchi, 2007; Itoh et al., 2010; Jiang et al., 2005; Kim and Snider, 2011; Lewis et al., 2013). The ability of GSK3β to crosstalk with most of the pathways reported to control these morphological mechanisms, at the transcription, translation and cytoskeleton level, suggests that it may function as a central node in the coordination and integration of neural development and the establishment and maintenance of polarity (Beurel et al., 2015; Guo et al., 2016; Hur and Zhou, 2010; Inoki et al., 2006; Kim and Snider, 2011).

The role of GSK3β in axonal growth has been demonstrated both at the developmental level (Hur and Zhou, 2010; Hur et al., 2011; Kim et al., 2006) and in regenerative processes following axonal injury (Diekmann and Fischer, 2015). In this regard, although the control of axonal growth has been long recognised (Kim and Snider, 2011), its precise role in regeneration has been more controversial (Leibinger et al., 2017), mainly due to the fact that a multitude of regulatory pathways and targets can be involved in GSK3β activity. In effect, the capacity of GSK to control such an array of cellular functions may arise from the multiple sophisticated mechanisms that regulate its action and protein expression, ensuring that it can only phosphorylate substrates at a precise time and in discreet subcellular compartments (Beurel et al., 2015).

As fundamental regulators of protein translation in the nervous system (Davis et al., 2015), the investigation of miRNAs and their specific role in the axon has rapidly expanded in recent years (Kaplan et al., 2013; Wang et al., 2015; Zhang et al., 2015). Studies from us and others (Bellon et al., 2017; Dajas-Bailador et al., 2012; Sasaki et al., 2014) have shown how regulation of local translation by specific miRNAs can control energy metabolism, growth and branching of axons. For example, Kar et al. (2013), elegantly showed how axonal transfection of miR-16 or a miR-16 inhibitor in rat sympathetic neurons was able to regulate mRNA levels of two of its targets (eIF2B2 and eIF4G2) in the axon, whereas no effect on the levels in the soma of the neuron was observed. In CNS neurons, miR-9-5p was shown to locally control axon development by targeting the microtubule-associated protein Map1b (Dajas-Bailador et al., 2012).

Here, we demonstrate that axonal miR-26a can modulate neuron polarity and axon growth in primary cortical neurons via the regulation of GSK3β protein levels. Inhibition of miR-26a led to a decrease in neurons with single axonal projections, while its overexpression promoted the generation of neurons with multiple ‘axon-like’ neurites. In a recent study, miR-338 was shown to have a role in neuronal placement and polarisation in the cortical plate, controlling neuronal polarity, migration and/or cortical placement cues (Kos et al., 2017a). In the case of miR-26a, we show a cell-autonomous role in the regulation of axon specification and growth of CNS neurons, a function that was also recently described for miR-140-3p (Ambrozkiewicz et al., 2018). In this study, miR-140-3p translationally inhibits the expression of the Src family tyrosine kinase Fyn to control laminar distribution and polarised morphology. However, unlike miR-140-3p, which serves to inhibit axon formation in developing cortical neurons, endogenous miR-26a promotes axon specification and growth in primary cortical neurons. In primary cortical neurons, the levels of miR-26a showed a trend towards a decrease from the time of plating, which stabilised towards later stages of culture (DIV 9), when fast axonal growth starts to decline and synaptic maturation begins (Chiappalone et al., 2006; Dotti et al., 1988; Opitz et al., 2002). *In vivo*, levels of miR-26a have been reported to increase from ∼E15 to P0 (Zhang et al., 2018), probably reflecting the active growth of axons during this period of cortical development (Lewis et al., 2013).

The unique ability of miR-26a to affect both polarisation and axonal growth is mainly achieved via the targeting of GSK3β, which is a known regulator of both processes. Additionally, the fact that miR-26a can also target PTEN, an upstream member of the GSK3β signalling pathway, reinforces its role as an important regulator of axon development. Crucially, our study has also unravelled a previously unknown mechanism for neuronal information processing and GSK3β signalling in developing CNS neurons. In effect, local inhibition of miR-26a in the axon produced a significant increase in GSK3β protein levels and a decrease in axonal growth. Although axonal inhibition of miR-26a increased GSK3β protein levels in both the axon and soma of cortical neurons, this was not observed when inhibition of this microRNA was restricted to the soma side of compartmentalised microfluidic chambers, indicating an axon-exclusive regulation of GSK3β translation via miR-26a.

Functionally, GSK3β activity is needed in both soma and axon compartments, as the decrease in axonal length observed after inhibition of miR-26a in the axon was prevented by local application of the inhibitor of GSK3 activity (SB415286) in either the soma or axon side of microfluidic chambers. The GSK3β expression studies reveal a molecular mechanism where local translation of GSK3β in the axon is normally repressed by the presence of miR-26a, thus allowing axonal development and growth. However, when miR-26a function is inhibited in the axon, local translation of GSK3β is triggered, followed by transport to the soma of cortical neurons. Although the activity of GSK3β is required in the axon and soma, the somatic increase in GSK activity that is capable of regulating axon function is dependent on its translation in the axon compartment. Confirmation of this mechanism was first provided by the inhibition of local translation (emetine addition), which blocked the increase in GSK3β levels in both axon and soma; and, second, by the impairment of axonal transport (nocodazole application), which prevented the increase in GSK3β protein levels that was observed in the soma after axonal inhibition of miR-26a. Reassuringly, nocodazole application did not stop the miR-26a inhibition-dependent local translation of GSK3β in the axon, but the use of dynein inhibitors (i.e. ciliobrevin; Walker et al., 2018) would be needed to confirm the specific molecular mechanism involved in its retrograde transport to the soma. Levels of GSK3β protein after the concurrent inhibition of miR-26a in the axon and impairment of axonal transport indicates that 6 h are sufficient to produce a significant increase in GSK3β protein levels in the axon. This likely reflects the high rate of GSK3β axon translation and transport following release of miR-26a repression.

The lack of effect when a miR-26a inhibitor or GSK-TSB are applied only in the soma suggests that the interaction of miR-26a with Gsk3b mRNA takes place exclusively in the axon compartment, possibly reflecting independent transport of the precursor miRNA, as recently shown by Corradi et al. (2018). In the case of GSK3β, its activity was previously shown to regulate axon regeneration in a coordinated way, acting at the growth cone to control microtubule dynamics and in the neuronal soma to control gene expression (Saijilafu et al., 2013). In follow-up studies, the Zhou group (Jiang et al., 2015) proposed that miR-26a activity in peripheral neurons was needed to maintain lower protein levels of GSK3β and efficient axon regeneration, via a mechanism where miR-26a did regulate GSK3β exclusively in the soma, upstream of the regeneration-associated transcription factor Smad1. Our findings in CNS developing neurons demonstrate a previously unforeseen functional link, where actions of miR-26a in the axon can control GSK3β levels and activity locally, but also be retrogradely transported to impact on somatic regulatory mechanisms that control axon development and growth. It is possible to speculate that Smad1 might also be acting downstream of miR-26a and GSK3β in the soma of CNS neurons, as demonstrated by Jiang et al. (2015) in peripheral axons. Whether the retrograde mechanism observed by us in cortical cultures is also relevant in sensory neurons remains to be established.

In recent years, local protein synthesis has been confirmed as a cellular process that can provide structural and regulatory components that are specifically needed in the axon, either during development, synaptic maturation or regeneration (Batista et al., 2017; Batista and Hengst, 2016; Costa and Willis, 2018; Campbell and Holt, 2001; Si et al., 2003; Verma et al., 2005; Yoon et al., 2012). Among the least expected members of the axon translatome are transcription factors (Cox et al., 2008; Ji and Jaffrey, 2012) such as CREB, which can be retrogradely transported to the nucleus to promote neuronal survival. In this way, local axon translation can facilitate and amplify communication between the axon and the neuronal soma, allowing the transport of newly synthesised axon ‘protein messengers’ (Cox et al., 2008). Although this mechanism has been shown in peripheral neurons and after injury (Ben-Yaakov et al., 2012; Terenzio et al., 2018; Walker et al., 2018), we show that retrograde transport of a locally translated signalling molecule can achieve a functional outcome in developing CNS neurons. Additionally, our results demonstrate how a single miRNA can use the spatiotemporal control of axonally originated protein synthesis to impact events globally in the soma. This is a significant observation that challenges the prevalent view of miRNAs as only fine tuners of protein translation. In fact, localised regulation by specific miRNAs can dramatically change protein levels in defined neuronal compartments. The notable finding is how these changes in the axon can be used to communicate signalling information to the soma, in ways that can influence axonal growth.

Our findings have placed miR-26a at a junction of regulatory mechanisms that are able to impinge on neuronal polarity and axon development via the control of GSK3β levels. In this context, the relatively high levels of miR-26a expression in mature neuronal cultures and CNS raises potentially relevant questions about its role in adult brain. There is now a clear understanding of how the loss of axon and neuron connectivity constitutes a fundamental step in the early and progressive degradation of network information capacity (Coleman, 2005; Conforti et al., 2007). Interestingly, both miR-26a, as part of a signature group of miRNAs known to be deregulated in Alzheimer's disease (Cogswell et al., 2008; Leidinger et al., 2013), and GSK3β, which has shown increased activity leading to tau hyperphosphorylation in various Alzheimer's disease models, have been implicated in neurodegenerative processes (Dargahi et al., 2015; Hooper et al., 2008). Future work will need to establish whether the spatiotemporal control of GSK3β molecular mechanisms that are regulated by axonal miR-26a in developing neurons could also have an impact in neuronal function in the mature and ageing brain.

## MATERIALS AND METHODS

### Primary cortical neuron cultures

Mice (C57/BL6) were housed and bred in compliance with the ethics and animal welfare in accordance to the Animal (Scientific Procedures) Act 1986. C57/BL6 mouse embryos at E16.5 stage of development were culled and their brains removed. Brain cortices were dissected, and the meninges separated under a dissection microscope. The tissue was further incubated in Hanks Balanced Salt Solution (HBSS, Ca^2+^ and Mg^2+^-free; Gibco) with 1 mg/ml trypsin and 5 mg/ml DNAse I (Sigma-Aldrich) at 37°C for 30 min. Following the addition of 0.05% (v/v) soybean trypsin inhibitor (Sigma-Aldrich), the tissue was mechanically dissociated in Neurobasal media (Invitrogen) supplemented with 1× GlutaMax and 2% B-27 (Gibco). Dissociated neurons were resuspended in supplemented Neurobasal media (10×10^6^ cells/ml). For functional assays and RNA extraction, neurons were plated at a final seeding density of 1.75×10^5^ cells/cm^2^ in six-well plates (Corning) with or without 22×22 mm glass coverslips (Menzel Glaser) previously coated with 50 µg/ml poly-L-ornithine (PLO; Sigma-Aldrich). For functional assays with cell-permeable target site blockers (Table S1), neurons were plated at a final density of 3.5×10^3^ in 12-well plates (Corning) with PLO-coated 19 mm glass coverslips (Menzel Glaser). For experiments that required over 7 days in culture, media was replenished with one quarter of its volume every 2-3 days.

Depending on the experimental approach, neuronal transfections were performed 4 h, 24 h or 5 days after plating using 5 µl/well of Lipofectamine 2000 reagent and 250 µl/well of Opti-MEM reduced-serum media (Thermo Fisher Scientific), in accordance to manufacturer's instructions. miRCURY LNA (Locked Nucleic Acid) microRNA inhibitor (50 nM), inhibitor control (50 nM), mimic (20 nM) and mimic control (20 nM) of miR-26a (all Qiagen) were used for transfections. In all cases, 1 µg pmaxFP-Green (Lonza) was co-transfected for visualisation of transfected neurons. For protein overexpression studies, neurons were transfected with 1 µg pmaxFP-Green (Lonza; hereafter referred to as GFP) and either 1 µg of empty vector or 1 µg of pcDNA-GSK3β.

To rescue the effects of miR-26a inhibition, cortical neurons were co-transfected with 1 µg GFP and LNA inhibitor control or LNA miR-26a inhibitor (50 nM), while the GSK3 inhibitor SB415286 (Tocris) was used at a concentration of 1 µM and added to the culture 24 h after plating. Cell-permeable target site blockers (Table S1) GSKβ-TSB-1 (50 nM) and GSKβ-TSB-2 (50 nM) (hereafter referred to together as GSKβ-TSB for simplicity) or GSKβ-TSB-control (100 nM) were applied 24 h after plating and incubated for 48 h.

In all experiments, cortical neurons were fixed in 4% paraformaldehyde (Thermo Fisher Scientific) 72 h after transfection and washed in PBS before direct visualisation and/or immunostaining. Microscope imaging was carried out using a wide-field fluorescence microscope (Axiovert 200M, Zeiss) coupled to a CCD camera (Photometrics CoolSnap MYO) and Micro-Manager software 1.4.21 (Edelstein et al., 2010).

### Neuro2A neuroblastoma cell line cultures

Neuro2a cells (ECACC; Accession Number 89121404) were maintained in Dulbecco's modified Eagle's medium (Sigma-Aldrich) supplemented with 10% foetal calf serum and 1% penicillin-streptomycin (Sigma-Aldrich) and seeded onto 12-well plates (Corning) at a density of 1.5×10^5^ cells/well. After 24 h, miR-26a inhibitor (100 nM), inhibitor control (100 nM), miR-26a mimic (100 nM) and mimic control (100 nM) were transfected using a 1.5 µl/well of Lipofectamine 2000 reagent and a 200 µl/well of Opti-MEM. Media were changed 24 h later and cells scraped into 100 μl of RIPA buffer [50 mM Tris-HCl (pH 8.0) with 150 mM sodium chloride, 1% NP-40, 0.5% sodium deoxycholate and 0.1% sodium dodecyl sulfate; all Sigma-Aldrich] with protease inhibitor cocktail (1:100, Sigma-Aldrich) 48 h after transfection. Samples were then lysed by snap freezing and centrifuged for 20 min at 12,000 ***g*** to collect the supernatants.

### Western blotting

Neuro2A protein extracts were run on a 12% SDS-PAGE gel, blotted onto a 45 µm nitrocellulose membrane (GE Healthcare) and blocked in 5% skimmed milk in Tris-buffered saline 0.1% Tween (TBS-T; Sigma-Aldrich) for 1 h at room temperature. Membranes were probed overnight with anti-GSK3β and anti-GAPDH (Table S1) in 5% skimmed milk in 0.1% TBS-T followed by a chemiluminescence protocol using HRP-conjugated rabbit anti-mouse Ig (Dako; 1:3000). After a 1 min membrane incubation with Western Lightning Plus ECL (Perkin Elmer), signal detection was performed with Amersham Hyperfilm ECL (GE Healthcare) in a dark chamber. Image analysis was conducted using the peak area method for relative quantification in Fiji software and GSK3β signal was normalised to the loading control (GAPDH). Data are expressed as relative density to control of two independent experiments.

### Compartmentalised neuronal culture in microfluidic chambers

Primary cortical neurons were cultured for 5 days in microfluidic devices with 150 µm long microgrooves (SND150; Xona Microfluidics). The use of these chambers allows the fluidic isolation and functional compartmentalisation of the axon and somatodendritic compartments. For simplicity the somatodendritic compartment is hereafter designated somal channel throughout the text. The devices were prepared as described previously (Garcez et al., 2016). Briefly, ethanol sterile devices were mounted onto PLO-coated 35 mm culture dishes (Nunc, Thermo Fisher Scientific) and both channels were equilibrated for 1 h with supplemented Neurobasal media. Following collection of excess media from the devices' reservoirs, cortical neurons were added onto the designated somatodendritic compartment at a seeding density of 4×10^6^ cells/ml and incubated for 30 min (37°C 5% CO_2_) to allow for cell attachment. The reservoirs of the devices were then topped up with supplemented Neurobasal media and incubated at 37°C in 5% CO_2_. Axons were allowed to extend and cross the microgrooves to the axonal channel. Even after 8 days of culture, only axonal projections have grown long enough to extend through microgrooves into the opposite side of the channel. This is demonstrated by the fact that, at this stage of culture, dendritic staining with MAP2 was restricted solely to the somatodendritic compartment of microfluidic chambers (Fig. S8B). Moreover, average dendritic length at this stage of culture in our experimental model was 91.53±2.7 µm, significantly shorter than the 150 µm long microgrooves of the chambers.

Functional experiments were performed after 5-6 days *in vitro.* Cell-permeable Power inhibitor miR-26a or Power inhibitor control at 100 nM (all Qiagen) and cell-permeable GSK3β-TSB [GSKβ-TSB-1 (50 nM) plus GSKβ-TSB-2 (50 nM)] or GSK3β-TSB-control at 100 nM was added to the axon side of the microfluidic device at day 5. As cell-permeable oligonucleotides are incorporated by non-assisted uptake, higher oligo concentrations are necessary due to the inherently less efficient uptake kinetics. A difference in volume of ∼100 µl was maintained at all times between the somal and axonal channels in order to maintain fluidic isolation. Live imaging of the axons in the axonal channel was performed at different time points (0, 24 and 48 h) after addition of inhibitors using an Axiovert 200 M microscope (Zeiss) with a 10× phase contrast lens. When required, the axons projecting into the axonal chamber were labelled with either acetylated tubulin (Fig. 5) or βIII-tubulin (Figs 6–8). To rescue the local effects of miR-26a inhibition, we added the GSK3 inhibitor SB415286 (1 µM) to the axonal channel, together with the cell-permeable inhibitor of miR-26a-5p or inhibitor control at 100 nM. Live imaging of the axons in the axonal channel was performed at 0 h and 24 h after addition of drugs/inhibitors. To impair axonal transport, nocodazole (100 nM, Sigma-Aldrich) was added 18 h after the addition of the cell-permeable miR-26a inhibitor. To inhibit protein translation, emetine (100 nM, Sigma-Aldrich) was added 18 h after the addition of cell-permeable miR-26a inhibitor. Following 6 h of nocodazole or emetine incubation (24 h in total after addition of miRNA inhibitors/controls), devices were removed, and neurons fixed and immunolabelled for GSK3β protein and βIII-tubulin.

### Mitochondrial motility

To test the capacity of nocodazole to disrupt axonal transport, neurons were plated in PLO-coated 35 mm high µ-Dishes (Ibidi) at a seeding density of 3.5×10^3^/ml cells, treated at day 5 with 100 nM nocodazole or DMSO for 6 h. MitoTracker Green FM (Invitrogen) was then incubated at 100 nM for 30 min at 37°C. Images of axons were acquired at 37°C on a Zeiss TIRF microscope coupled with an EMCCD (Photometrics PVCam) camera using ZEN 2010 software (Carl Zeiss), at the rate of 1 frame/s for 3 min. Videos of 15-25 random axon fields were acquired from three independent experiments, and motile and static mitochondria were scored using Fiji software. Data are expressed as a fraction of motile or static mitochondria from total mitochondria (mean±s.e.m.).

### DNA constructs and oligos

For the pcDNA-GSK3β and pcDNA-PTEN constructs, both *Gsk3b* and *Pten* cDNAs were PCR amplified from a replication construct [pMD18-TSimple (Sino Biological) and pCMV-Sport6, respectively (Source Biosciences)], with primers containing the appropriate restriction sites (Table S1, IDT). The amplicons were cloned into pcDNA3.1/Zeo(+) vector (a kind gift from Dr Simon Dawson, University of Nottingham, UK), using Nhe/XbaI (*Gsk3b*) and BamHI/XbaI (*Pten*) restriction sites. All miRNA mimics, inhibitors, cell-permeable Power inhibitors, target site blockers (GSK3β-TSB-1, GSK3β-TSB-2 and GSK3β-TSB-control) and miRNA qPCR primers used in this study were obtained from Qiagen and are listed in Table S1, together with mRNA qPCR primers.

### RNA extraction

On six-well plates, cells were scraped into 250 µl/well of TRIzol Reagent (Thermo Fisher Scientific). Axonal RNA was obtained as described previously (Garcez et al., 2016) with a few modifications. Cortical neurons were grown in microfluidic chambers for 8 days, when the average dendrite length is ∼40% lower than the 150 µm microgrooves (91.53±2.78 µm) and Map2 staining shows no crossover of dendritic projections into the axonal side (Fig. S8B).

Cells were washed twice with PBS before addition of 20 µl of TRIzol to each reservoir of the axonal channel and incubation for 2 min at room temperature. A volume of 100 µl of PBS was kept in the soma reservoirs to prevent contamination from opposite channel. Following collection of axonal sample, the somatodentritic fraction was obtained in the same manner. Fractions from 40-50 devices were collected for each independent experiment. Total RNA was isolated following manufacturer's instructions and resuspended in RNAse-free water (Thermo Fisher Scientific) before storage at −80°C.

### RT-qPCR

For miRNA expression studies, cDNA was synthesised from mature miRNAs using the miRCURY LNA Universal cDNA synthesis kit (Qiagen, UK) according to the manufacturer's instructions, using 10 ng of total RNA. For each timepoint, five biological samples were run in duplicate using miRCURY LNA primers (Table S1, Qiagen, UK). RT-qPCR was undertaken using the ExiLENT SYBR Green master mix kit (Qiagen, UK). For mRNA targets, cDNA was synthesised from 100 ng total RNA (five biological replicates), using SuperScript IV and Oligo(dT)_20_ primer (Invitrogen) according to the manufacturer's instructions. qPCR was undertaken using the PowerUp SYBR Green (Applied Biosystems) using 1.5 µl cDNA per replicate and 400 nM primers (Table S1, IDT). In both cases, PCR amplification was carried out in the Applied Biosystems Step One Plus thermocycler, using cycling parameters recommended by Qiagen (miRNA) and Applied Biosystems (mRNA). Data were acquired with Applied Biosystems SDS2.3 software. Passive reference dye ROX (Thermo Fisher Scientific) was included in all reactions. Expression data were analysed by relative quantification using the comparative Ct method (2^−ΔΔCt^). miR-26a-5p levels were analysed as relative expression to 4 h, using the geometric mean of miR-100-5p, miR-128-3p, miR-134-5p, miR-434-3p and let7a-5p as a reference. miRNA reference genes were selected according to two parameters: detectable expression by RT-qPCR in axonal RNA samples; and stable expression in previous in-house RT-qPCR studies on the development of cortical neuronal cultures. All of the selected miRNA reference genes changed less than 1 Ct (*n*≥3) over 12 days in culture (Table S2). In studies of dissociated cortical cultures, *Gsk3b* levels were analysed as relative expression to 4 h, where the geometric mean of *Gapdh* and *Ube2* levels was used as reference. All data are expressed as fold change to 4 h±s.e.m. RT-qPCR experiments in the axonal fractions of cortical primary microfluidic cultures showed detectable levels of miR-26a and GSK3β in cortical axons (miR-26a average Ct=29.95±0.05; GSK3β average Ct=26.92±0.30; *n*=3 independent experiments). miR-26a levels in the axons were within the range of detection for mature miRNAs and comparable with previous miRNA qPCR quantification experiments in cortical and DRG axons (Natera-Naranjo et al., 2010; Zhang et al., 2013).

### Immunofluorescence

Cortical neurons cultured on coverslips or microfluidic devices were fixed using 4% paraformaldehyde (w/v) (Thermo Fisher Scientific) for 30 min, washed with 10 mM glycine in PBS, permeabilised in PBS/glycine-Triton (1× PBS, 10 mM Glycine, 0.2% Triton X-100; Sigma-Aldrich), blocked with 3% bovine serum albumin in PBS (BSA; Sigma-Aldrich) and further incubated with the appropriate primary antibodies overnight (Table S1). Following PBS-Triton 0.1% washes, cells were incubated with secondary antibodies (Alexa Fluor 488 and 568; 1:300 Molecular Probes) and mounted with Vectashield Hardset mounting media with Dapi (Vectorlabs).

### Data analysis

#### Measurement of axons in dissociated cortical cultures

For quantification of axon length, an axon was defined as a neurite that was at least three times the length of any other neurite and measured from the soma to the distal extent of the central region of the growth cone using Fiji software (Dajas-Bailador et al., 2008; Schindelin et al., 2012). Data are expressed as mean percentages of respective controls (∼300 axons measured for each condition from four to six independent experiments)±s.e.m. In GSKβ-TSB experiments, cultures were immunostained for βIII-tubulin and ∼100 neurons from ∼40 random fields were analysed per condition per experiment (∼400 axons from four independent experiments). The average length of axons in control groups was 115±1.96 μm (mean±s.e.m.).

#### Measurement of neurites and developing dendrites length

In experiments where neurons were transfected with 20 nM of miR-26a mimic, neurite length was assessed by measuring the length from the soma to the distal tip of all the projections in each GFP-positive cell. In miR-26a inhibitor transfection experiments (50 nM), all neurites of non-polar neurons and developing dendrites of single-axon (polarised) neurons were measured following the same protocol. Data are expressed as mean percentages of respective controls (∼700 projections measured for each condition from four independent experiments)±s.e.m.

#### Polarity assessment

The aforementioned criteria for the definition of axon was also used to define a neuron as a polarised cell. Neuronal polarisation in culture was then assessed by determining the fraction of polarised cells with respect to the total number GFP-positive cells. The dataset of five independent experiments was normalised and expressed as percentage of control (mean±s.e.m.). Multi-polar neurons were identified as neurons bearing more than one axon, defined as a neurite with JIP1-positive tips (Dajas-Bailador et al., 2014, 2008; Deng et al., 2014; Fu and Holzbaur, 2013). The data set of five individual experiments was normalised to respective control and expressed as percentage of control (mean±s.e.m.).

#### Quantification of fluorescence signal

Neurons labelled for GSK3β were imaged at 63× and images were further processed with Fiji software. Somas and growth cones were manually selected and the area, mean grey value and integrated density were measured. In order to correct for background in each image, three empty areas were selected around every soma/growth cone. Total cell fluorescence (C.F.) per cell was calculated as the measured integrated density corrected for background, according to the formula: C.F.=Integ.Density−[Area of soma×Average (mean grey value of background)]. For quantification of endogenous GSK3β in culture, ∼200 somas and 100 growth cones were measured in each condition from at least four independent experiments. Data were normalised to the average C.F. of the control expressed in percentage as mean±s.e.m. To control for imaging artefacts, neurons labelled for βIII-tubulin were imaged following the same protocol in both neuronal somas and growth cones (Fig. S7).

#### Measurement of axon length in microfluidic cortical cultures

The length of the axons was measured using Fiji software by tracing at least 125 axons in each condition from five independent experiments; each axon was traced from the edge of microgrooves to the growth cone of the longest axonal branch. In all the experiments, data for the different timepoints in each chamber was normalised to t0 and expressed as a percentage of respective controls (mean±s.e.m.).

#### Quantification of fluorescence signal in microfluidic cortical culture and after disruption of axonal transport

For quantification of endogenous GSK3β levels, ∼200 somas and ∼200 growth cones were measured in each condition from at least five independent experiments. Data were normalised to the average C.F. of the control expressed in percent as mean±s.e.m.

### Statistical analysis

In all statistical tests, *n* refers to the number of independent experimental repeats, which varied from four to eight depending on experimental model (see specific section for details). Data analysis was carried out using Prism v7.0 (GraphPad Software) and all data groups shown are expressed as mean±s.e.m. For sample sizes of *n*<5, individual data points are also shown in graphs. The probability distribution of the data set was analysed before further statistical analysis (Shapiro–Wilk test). Statistical evaluation between two groups was performed using unpaired Student's *t*-test. Analyses of more than two groups were carried out using one-way ANOVA with Bonferroni post-hoc analysis. Kruskal–Wallis' test followed by a Dunn's multiple comparisons test was used for non-parametric distributions. For all tests, *P<*0.05 was used as threshold for significant difference. For all tests, *P* values are two-tailed.

## Supplementary Material

Supplementary information
