# Supplementary material for: Spatiotemporal regulation of GSK3β levels by miRNA-26a controls axon development in cortical neurons
Source: Development. 2020 Feb 3;147(3):dev180232. doi: 10.1242/dev.180232 (PMC7033742; doi:10.1242/dev.180232)
Supplement: Supplementary information [file develop-147-180232-s1.pdf]

Figure S1

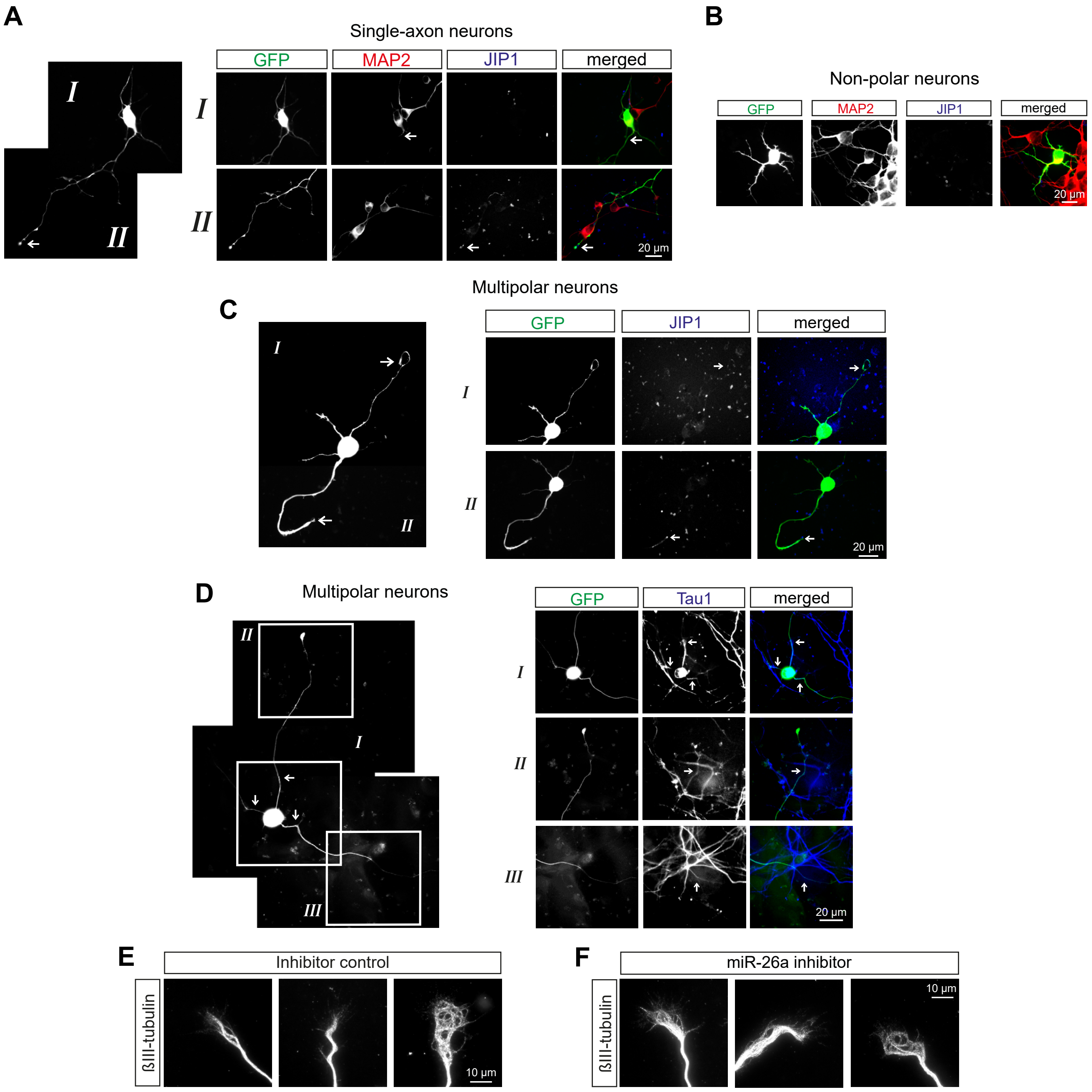

Fig S1. **Identification of neuron projection identity. Comparison between the dendrite marker MAP2 and the axonal markers JIP1 and tau**. (**A**) Representative image of a single-axon neuron, where MAP2 is absent from JIP1 positive neurite. (**B**) Representative image of non-polar neuron where JIP1 is absent from MAP positive neurons (**C**) Representative neuron with multiple axon-like JIP1 positive neurites. (**D**) Representative neuron with multiple axon-like Tau positive neurites. (**E-F**) Representative images of high magnification growth cones after addition of non-targeting controls [50 nM] or miR-26a inhibitor [50 nM], showing that there is no detectable difference in cytoskeleton structures, as labelled by βIII-tubulin.

Development • Supplementary information

Figure S2

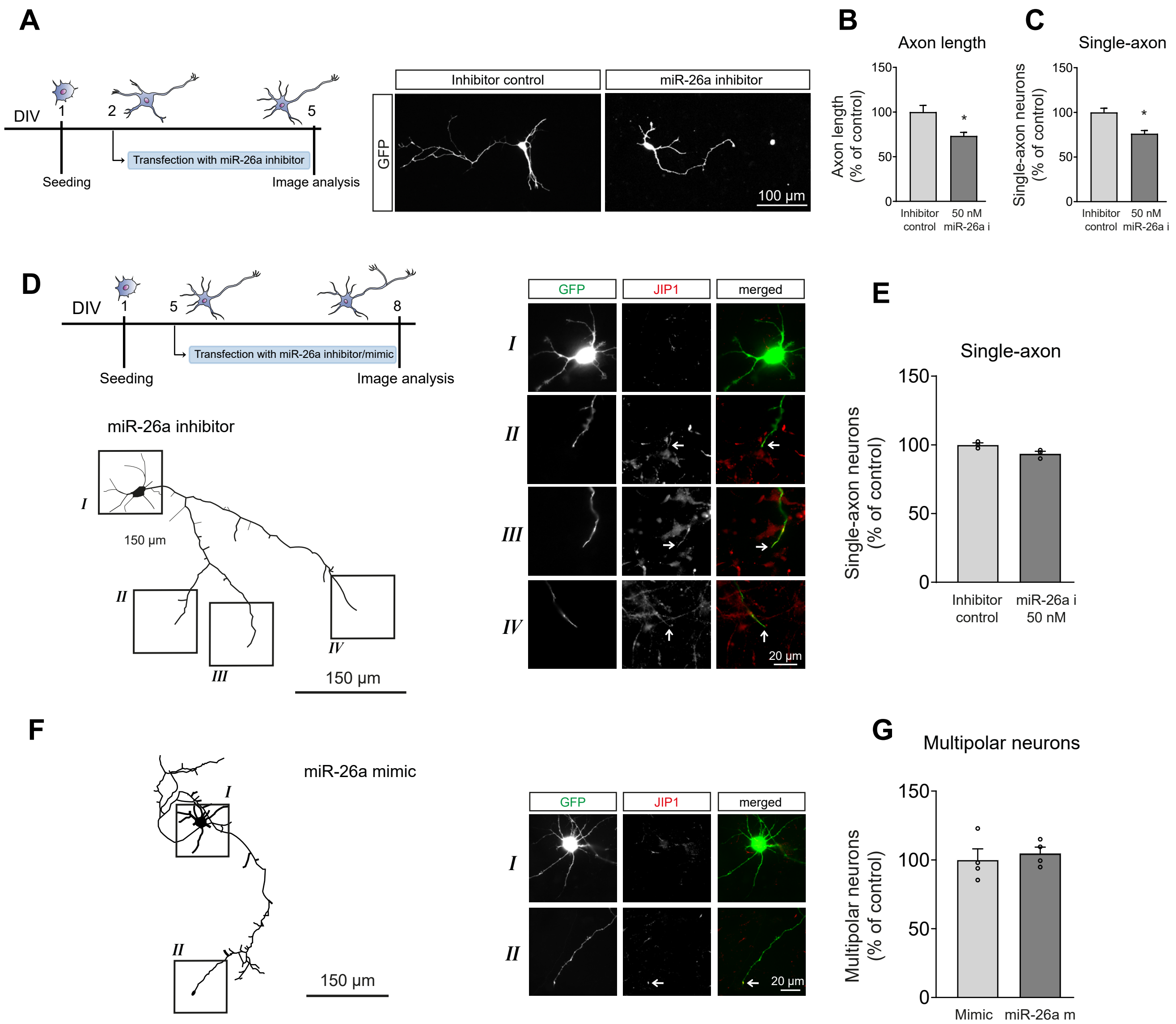

Fig. S2. **Temporal control of miR-26a in neuronal polarity**. (**A**) Diagrammatic representation of the experimental design used in B and C. Representative images of cortical neurons after transfection with GFP plus a miR-26a inhibitor 24 h after plating. (**B-C**) Quantification of axon length and number of single-axon neurons after inhibition of miR-26a (50 nM miR-26a i), showing up to 20 % decrease for both metrics compared to non-targeting controls, *n*=5. (**D**) Diagrammatic representation of the experimental design used in E-G and trace of a representative neuron after miR-26a inhibition at DIV5. Squares (*I-IV*) on the trace correspond to images from soma and neurite terminals of cortical neuron. Arrows indicate JIP1 labelling, which was used as a marker of axonal growth cones. (**E**) Quantification of the number of single-axon neurons after inhibition of miR-26a, expressed as a percent of neurons transfected with non-targeting control, *n*=4. (**F**) Representative traces of a neuron after over-expression of miR-26a at DIV5. Squares (*I-II*) on the trace correspond to images from soma and neurite terminals of cortical neuron. Arrows indicate JIP1 labelling, which was used as an established marker of axonal growth cones. (**G**) Quantification of the number of neurons with multiple axon-like processes after over-expression of miR-26a and expressed as a percent of neurons transfected with a non-targeting control, *n*=4. Data is expressed as mean ± SEM; Student's t-test: *: $p < 0.05$

Figure S3

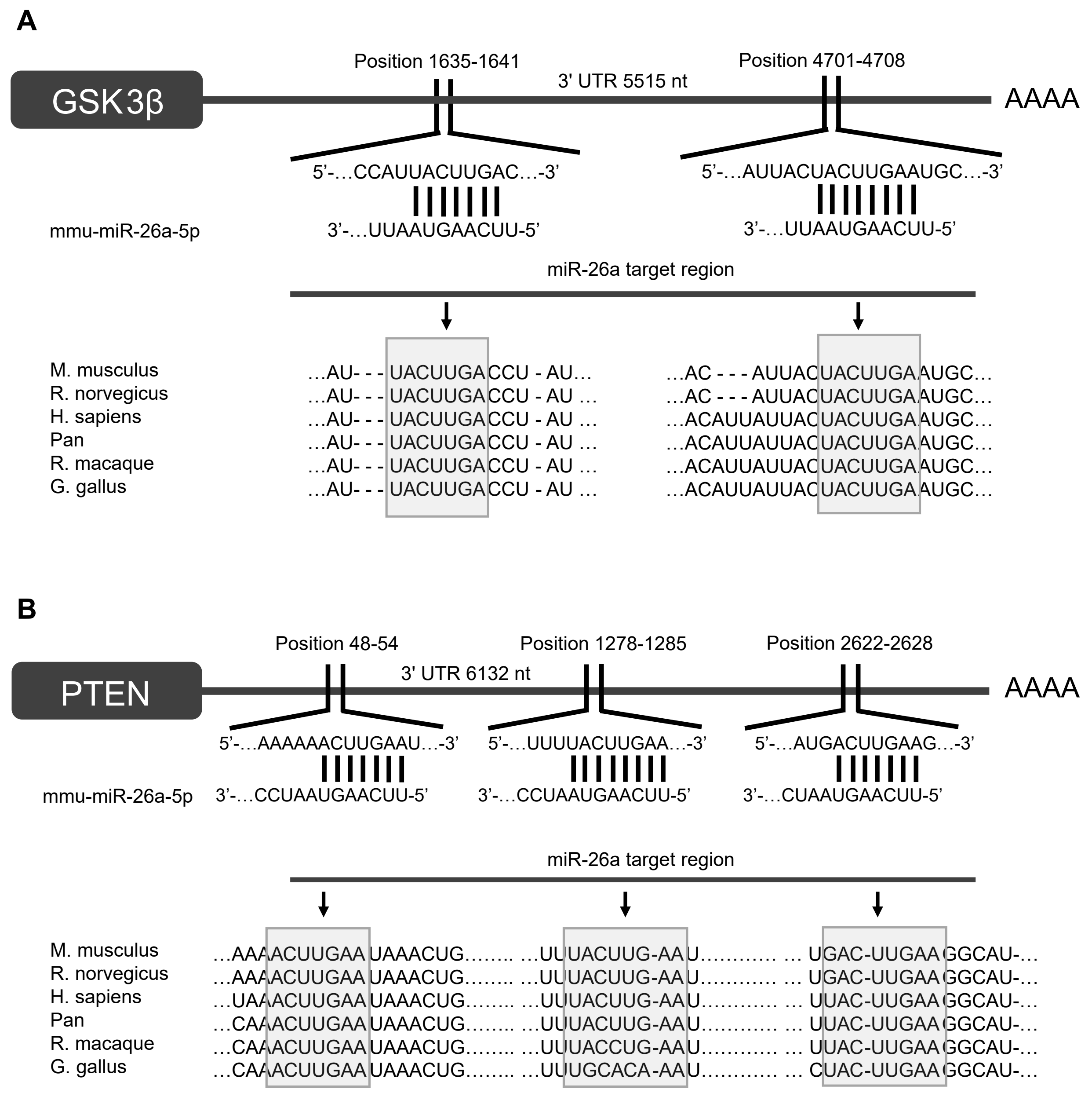

Fig. S3. **Conservation of miR-26a binding sites in *Gsk3b* and *Pten* 3'UTRs.** (**A**) Diagram of miR-26a sites within the 3'UTR of *Gsk3b* predicted by TargetScan, showing the complementary binding to miR-26a seed sequence and the conservation of miR-26a binding site across vertebrates. (**B**) Diagram of miR-26a sites within the 3'UTR of *Pten* predicted by TargetScan, showing the complementary binding to miR-26a seed sequence and the conservation of miR-26a binding site across vertebrates.

Development • Supplementary information

Figure S4

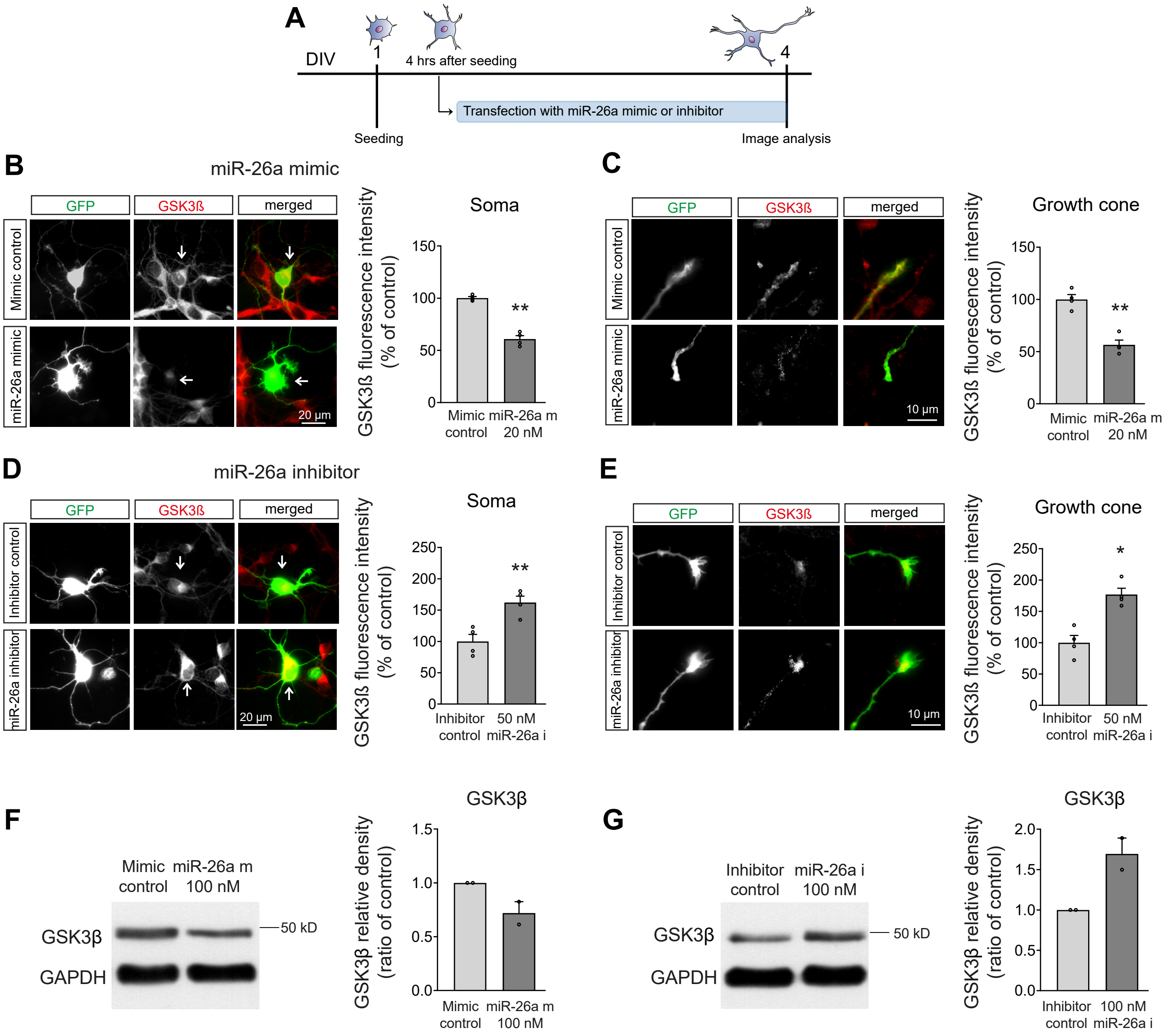

Fig S4. **miR-26a regulates the expression levels of GSK3β protein in neuronal somas and growth cones of single-axon neurons**. (**A**) Diagrammatic representation of the experimental design used in B-E. (**B**) Representative images of the soma of cortical neurons and (**C**) growth cones, after transfection with GFP plus a miR-26a mimic and immunostaining with GSK3β (red). Bar charts represent the quantification of GSK3β protein levels expressed as a percent of mimic controls, *n*=4. (**D**) Representative images of the soma of cortical neurons that have managed to develop a single-axon and (**E**) growth cones after transfection with GFP plus a miR-26a inhibitor and immunostaining with GSK3β (red). Bar charts represent the quantification of GSK3β protein levels expressed as a percent of non-targeting control, *n*=4. (**F**) Western blot of endogenous GSK3β and GAPDH proteins from Neuro2A cells after transfection with miR-26a mimic [100 nM] shows a decrease in GSK3β protein levels relative to mimic control, (**G**) whereas an increase is observed when miR-26a inhibitor [100 nM] is transfected in comparison to inhibitor control. Data are normalised to loading control (GAPDH) and presented as relative density to respective control. Data is expressed as mean ± SEM; Student’s t-test: *: p

Figure S5

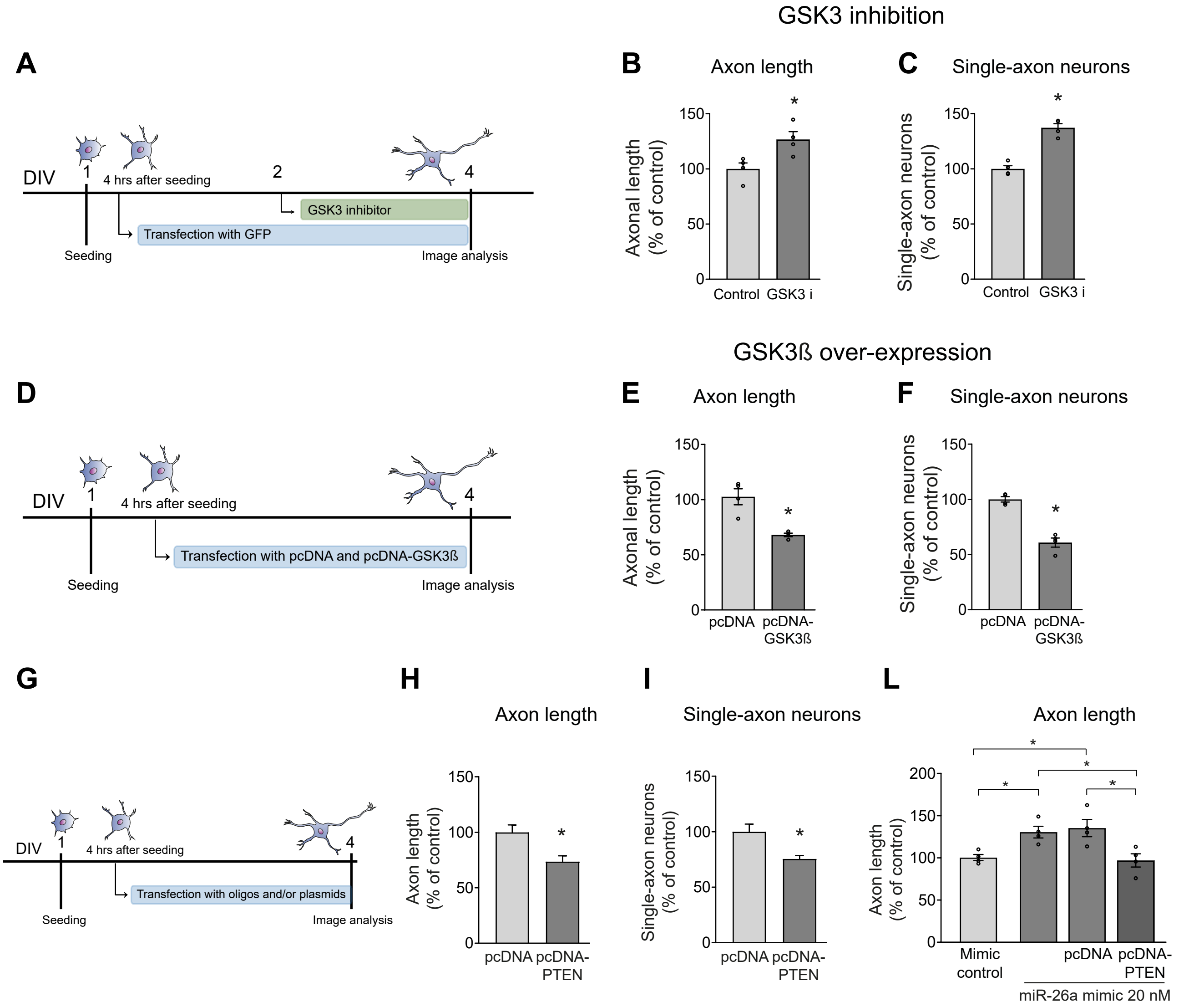

Fig. S5. **Both GSK3β and PTEN regulate neuronal polarity and axon outgrowth in primary cortical neurons**. (**A**) Diagrammatic representation of the experimental design used in B and C. (**B**) Quantification of axon length upon addition of GSK3 inhibitor (SB415286, 1 μM) 24 h after transfection with GFP, *n*=4. (**C**) Quantification of the number of single-axon neurons upon application of GSK3 inhibitor (SB415286, 1 μM) 24 h after transfection with GFP, *n*=4. (**D**) Diagrammatic representation of the experimental design used in E and F (**E**) Quantification of axon length after over-expression of GSK3β showing up to 25 % decrease compared to the empty vector. (**F**) Quantification of the number of single-axon neurons after transfection with GFP plus either empty vector or pcDNA-GSK3β, *n*=4. (**G**) Diagrammatic representation of the experimental design used in H-L (**H**) Quantification of axon length after over-expression of PTEN showing up to 20 % decrease compared to the empty vector *n*=5. (**I**) Quantification of the number of single-axon neurons after transfection with GFP plus pcDNA-PTEN, *n*=5. (**L**) Quantification of axon length after overexpression of both miR-26a and PTEN, *n*=4. Data is expressed as mean ± SEM; Student's t test (A-F), one-way ANOVA with Bonferroni's multiple comparisons post hoc tests (G): *: *p* <

Development • Supplementary information

Figure S6

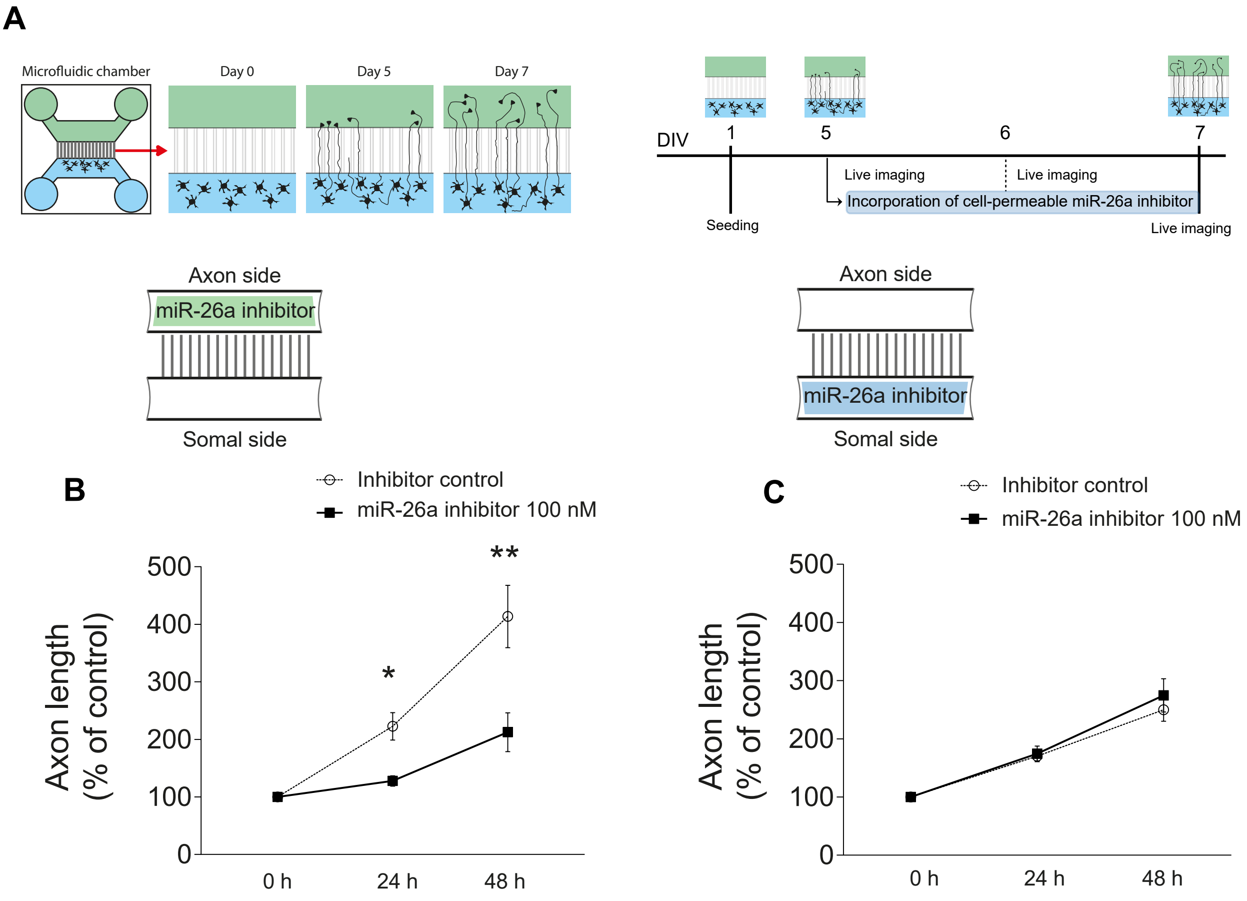

Fig. S6. **Localised inhibition of miR-26a in the axon can regulate axonal growth**. (**A**) Schematic diagram of a microfluidic device and the developmental growth of axons from primary cortical neurons and diagrammatic representation of the experimental design used in B and C. Cell-permeable miR-26a inhibitor or inhibitor negative control was applied to compartmentalised cultures at DIV5 and axons growing in the axonal side were measured at 0 h, 24 h and 48 h after application. (**B**) Inhibition of endogenous axonal miR-26a by application of a cell-permeable miR-26a inhibitor to the axonal compartment induced a decrease in axon length in comparison to a cell-permeable inhibitor negative control, 48 h after application. (**C**) When the cell-permeable miR-26a inhibitor or inhibitor negative control were applied to the somal compartment, no significant effect was observed in the length of axons growing in the axonal side. For all the experiments, schematics of the microfluidic chambers (upper left corner) depict where drugs were added. Application to the axon and the soma side is illustrated in green and blue, respectively. Data normalised to axon length at T=0 h (t0) and presented as percentage of t0, n=5. Data is shown as mean±SEM; one-way ANOVA with Bonferroni pots-hoc test, *: $p < 0.05$, **: $p \leq 0.01$.

Figure S7

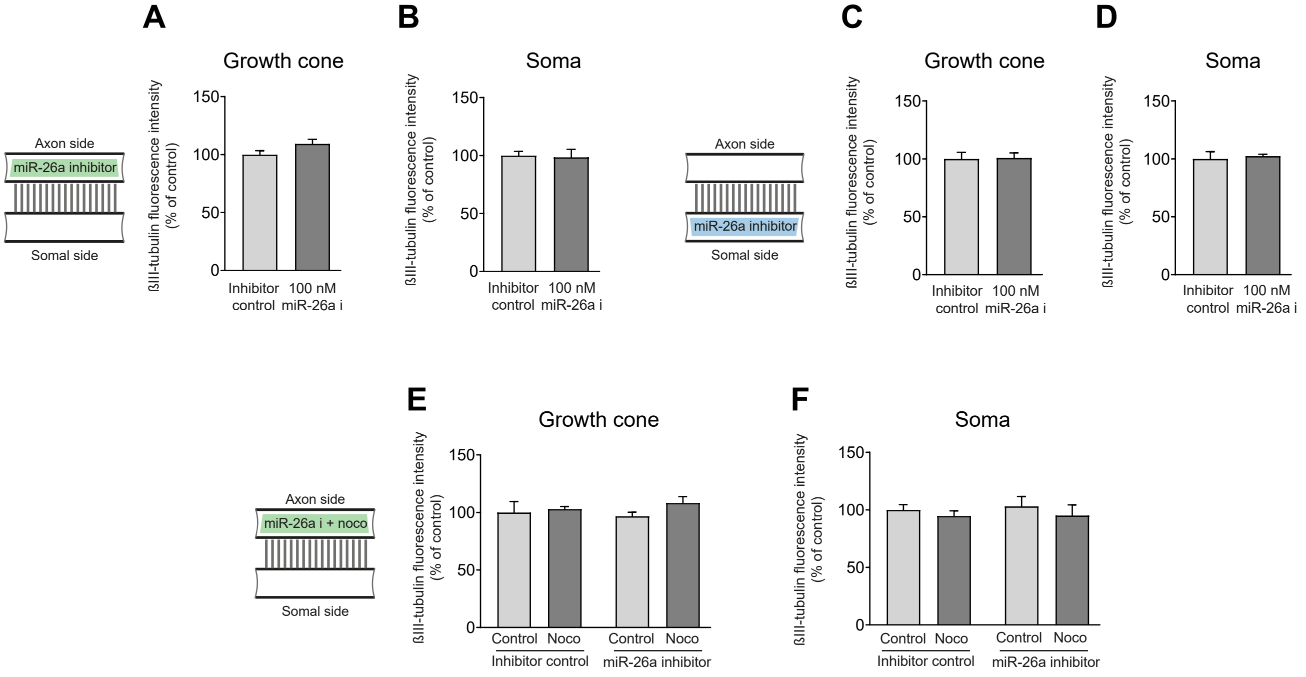

Fig. S7. **Levels of βIII-tubulin do not change after inhibition of miR-26a in different subcellular compartments**. Analysis of βIII-tubulin immunofluorescence levels in the growth cones and soma of cortical neurons after addition of miR-26a inhibitor [100 nM] in the (**A**-**B**) axon or (**C**-**D**) soma side of microfluidic chambers, *n*=5. (**E**-**F**) Analysis of βIII-tubulin immunofluorescence levels in the growth cones and soma of cortical neurons after addition of miR-26a inhibitor [100 nM] and nocodazole [100 nM] in the axon side of microfluidic chambers, *n*=5. Data is expressed as mean ± SEM.

Development • Supplementary information

Figure S8

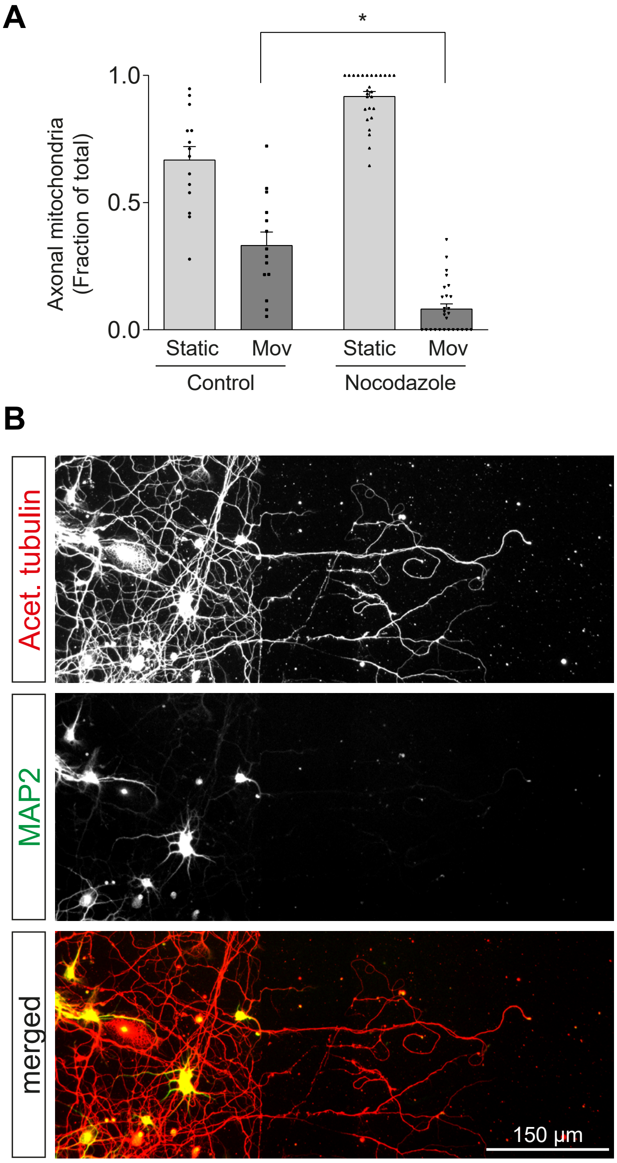

Fig. S8. (**A**) Quantification of mitochondria motility in axons of cortical neurons. Axons were labelled with the fluorescent marker mitotracker green, treated at DIV5 with either DMSO or 100 nM nocodazole and imaged 6 h later. Nocodazole application significantly decreased the number of moving mitochondria. Videos of 15-25 random axon fields were acquired from 3 independent experiments and motile and static mitochondria were scored using Fiji software. Data is expressed as mean ± SEM; Student's t-test: *: $p < 0.05$. (**B**) Representative images of cortical neurons cultured in microfluidic chambers for 8 days and immunolabelled with acetylated tubulin and MAP2, used as cytoskeletal and dendritic marker respectively. The panel shows that only axons extend through microgrooves and are able to reach the axonal compartment. Dendrites (MAP2 positive) are restricted to the somal compartment, as they are on average ~60 µm shorter than the 150-µm-long microgrooves. In our study the average dendrite length was 91.53 ± 2.7 µm.

Table S1. Reagents, primers and antibodies

PCR primers		
Gsk3b	F- 5’ CTC CAT TGG CTA GCT ATG TCG GGG CGA CCG AGA ACC ACC TCC TT 3’	
	R - 5’ GCG GTC TCT AGA TCA GGT GGA GTT GGA AGC TGA TGC AGA AGC 3’	
Pten	R - 5’ CTC CAT TGG GAT CCA TGA CAG CCA TCA TCA AAG AG 3’	
	R - 5’ GCG GTC TCT AGA TCA GAC TTT TGT AAT TTG TGA ATG 3’	
**mRNA qPCR primers**		
Gapdh	F - 5’ CTG CAC CAC CAA CTG CTT AG 3’	
	R - 5’ ACA GTC TTC TGG GTG GCA GT 3’	
Ube2	F - 5’ TGC CTG AGA TTG CTC GGA TCT 3’	
	R - 5’ TCG CAT ACT TCT GAG TCC ATT CC 3’	
Gsk3b	Cat no. MP200629, Sino Biological	
**miRNA qPCR primers**	**Target sequence**	**Qiagen catalogue no.**
miR-26a-5p miRCURY LNA miRNA PCR Assay	5'UUCAAGUAAUCCAGGAUAGGCU	Cat no.YP00206023
miR-100-5p miRCURY LNA miRNA PCR Assay	5'AACCCGUAGAUCCGAACUUGUG	Cat no.YP00205689
miR-128-3p miRCURY LNA miRNA PCR Assay	5'UCACAGUGAACCGGUCUCUUU	Cat no.YP00205995
miR-134-5p miRCURY LNA miRNA PCR Assay	5'UGUGACUGGUUGACCAGAGGGG	Cat no.YP00205989
miR-434-3p miRCURY LNA miRNA PCR Assay	5'UUUGAACCAUCACUCGACUCCU	Cat no. YP00205190
let-7a-5p miRCURY LNA miRNA PCR Assay	5'UGAGGUAGUAGGUUGUAUAGUU	Cat no. YP00205727
**miRNA functional assays**	**Target sequence**	**Qiagen catalogue no.**
miRCURY LNA miRNA Inhibitor Control A	TAACACGTCTATACGCCCA	Cat no.YI00199006
miRCURY LNA miR-26a-5p Inhibitor	GCCTATCCTGGATTACTTGA	Cat no.YI04102930
miRCURY LNA miRNA Mimic Control	UCACCGGGUGUAAAUCAGCUUG	Cat no.YM00479902
miRCURY LNA miR-26a-5p Mimic	UUCAAGUAAUCCAGGAUAGGCU	Cat no.YM00471417
miRCURY LNA miRNA Power Inhibitor Control A	TAACACGTCTATACGCCCA	Cat no.YI00199006-DDA
miRCURY LNA miR-26a-5p Power Inhibitor	GCCTATCCTGGATTACTTGA	Cat no.YI04102930-DDA
miRCURY LNA miRNA Power Target Site Blocker-1 (GSK3β-TSB-1)	GCATTCAAGTAGTAAT	Custom designed
miRCURY LNA miRNA Power Target Site Blocker-2 (GSK3β-TSB-2)	GTCAAGTAATGGCCCA	Custom designed
**Antibodies**	**Working dilution**	**Manufacturer details**
Anti-JIP1	1:100	Clone B-7, Cat no. sc25267; Santa-Cruz
Anti-GSK3β	1:100 IF/ 1:1000 WB	Clone 7, Cat no. 610201; BD Biosciences
Anti-MAP2	1:100	Cat. no. ab32454; Abcam
Anti-Acetylated tubulin	1:300	Clone 6-11B-1, Cat no. T7451; Sigma-Aldrich
Anti-βIII tubulin	1:100	Cat no. ab18207; Abcam
Anti-Tau-1	1:100	clone PC1C6, Cat no. MAB3420; MerkMilipore
Anti-GAPDH	1:5000	Cat. No. 60004-1-Ig: Proteintech

Table S2. miRNA reference genes expression levels in cortical cultures over development

	Average Ct values (n=3)				
**Day in culture**	**miR-100**	**Let-7a**	**miR-128**	**miR-134**	**miR-434**
Day 2	21.96022	18.97066	24.83843	32.24415	27.65191
Day 5	22.34609	18.85894	24.98995	32.36229	27.36239
Day 12	22.61753	18.83208	24.56682	31.92104	26.79716

Development • Supplementary information

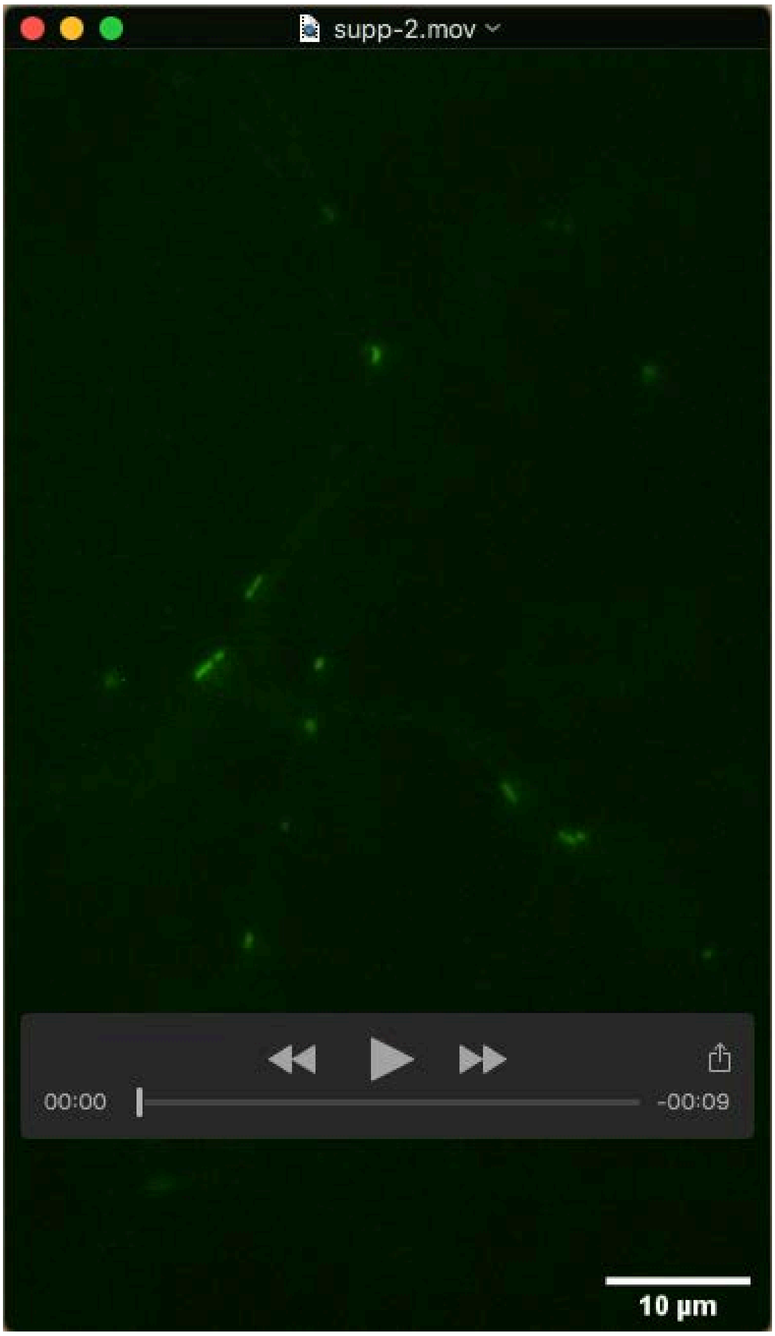

Movies 1. Mitochondria Motility in axons of cortical neurons labelled with MitoTracker green in control conditions. 1 frame/sec for 3 minutes

Development • Supplementary information

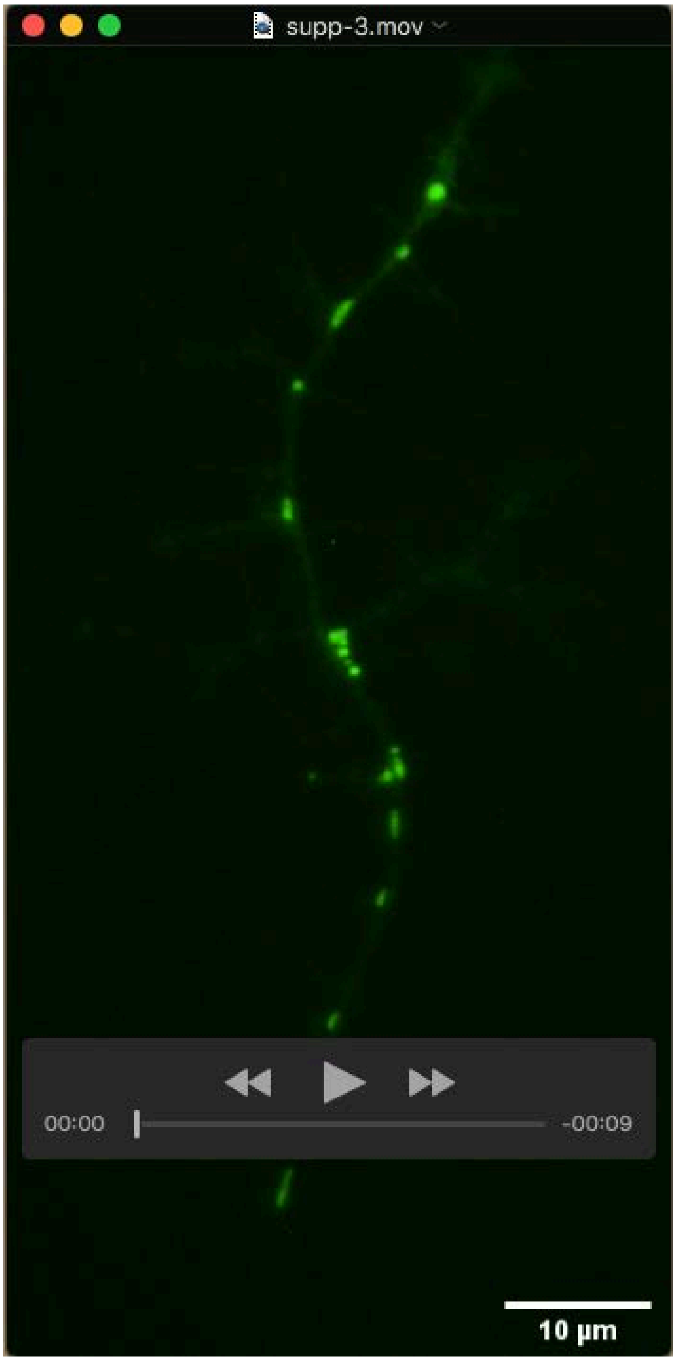

Movies 2. Mitochondria Motility in axons of cortical neurons labelled with MitoTracker green after Nocodazole application. 1 frame/sec for 3 minutes
